# A systematic review and meta-analysis of the effect of acupuncture therapy on the symptoms and immune indicators of ankylosing spondylitis

**DOI:** 10.3389/fneur.2025.1652356

**Published:** 2026-01-12

**Authors:** Yudi Wang, Jingyu Zhu, Furong Xue, Weiguo Liu

**Affiliations:** 1 College of Traditional Chinese Medicine, Shandong Second Medical University, Weifang, China; 2 Department of Traditional Chinese Medicine, Affiliated Hospital of Shandong Second Medical University, Weifang, China

**Keywords:** acupuncture, ankylosing spondylitis, immune factors, meta-analysis, systematic evaluation

## Abstract

**Background and objective:**

Ankylosing spondylitis (AS) is a chronic inflammatory disorder affecting sacroiliac joints, vertebral structures, paraspinal soft tissues, and peripheral joints. This study systematically investigated the mechanism of action of inflammatory factors underlying combined acupuncture therapy for AS, evaluating its impact on clinical manifestations and immune parameters.

**Methods:**

A comprehensive literature search was conducted across PubMed, Embase, Cochrane Library, SinoMed, CNKI, Wanfang, and VIP databases using keywords related to acupuncture and AS. Randomized controlled trials (RCTs) were screened using EndNote X9. The Cochrane RoB 2 tool, GRADE assessed methodological quality. Meta-analysis was performed in Stata 15.0, employing mean difference (MD), standardized mean difference (SMD), or relative risk (RR) with fixed- or random-effects models based on I^2^ heterogeneity. Publication bias was evaluated via funnel plots and Egger’s test, and subgroup analyses were conducted where applicable.

**Results:**

Fifty-two RCTs were included, of which 20 exhibited low risk of bias. Meta-analysis demonstrated that acupuncture, alone or combined with medication, significantly reduced pain (VAS: MD = −1.26, 95% CI [−1.44, −1.09]), inflammatory markers (CRP: MD = −3.49, [−4.12, −2.85]; ESR: MD = −5.36, [−6.82, −3.89]), and morning stiffness duration (MD = −1.32, [−1.87, −0.78]). Improvements were also observed in BASDAI, BASFI, IL-1, IL-6, IL-17, TNF-*α*, and IgA levels. Heterogeneity was moderate to high (*I*^2^: 59.70–90.00%). Subgroup analysis indicated that intervention design and treatment duration contributed to heterogeneity. No significant publication bias was detected for primary outcomes, though morning stiffness showed potential bias. Sensitivity analyses confirmed the robustness of inflammatory marker results.

**Conclusion:**

Acupuncture, particularly as an adjunct therapy, appears effective in alleviating clinical symptoms and reducing inflammatory activity in AS. However, high heterogeneity and variations in study design necessitate cautious interpretation. Further rigorously designed trials are warranted.

**Systematic review registration:**

https://www.crd.york.ac.uk/PROSPERO/view/CRD420251013146.

## 1 Introduction

Ankylosing spondylitis (AS) is a seronegative spondyloarthropathy characterized by idiopathic, chronic inflammation of the spine and sacroiliac joints (1, 2). The global prevalence of AS ranges from 0.1 to 1.4% (3), with a higher prevalence in young adult males approximately two to three times higher than in females (4). Clinical manifestations include lower back pain, morning stiffness, peripheral arthritis, and a limitation of spinal motion (5). In the advanced stages, ankylosing deformities of the spine, which possibly appear as bamboo-like changes (6), may result in severe impairment of spinal function and motion. Imaging modalities including radiography and magnetic resonance, are essential for early diagnosis of AS (7). Patients’ quality of life is substantially reduced by AS-related disability, imposing considerable economic and social burdens (8). AS involves (2, 9) genetic susceptibility (notably HLA-B27), immune dysregulation, gut microbiota, and mechanical stress. Its hallmark pathology is characterized by chronic inflammation triggered by activated inflammatory mediators, which in turn leads to an imbalance between bone formation and resorption (9, 10).

Current AS treatments encompass surgical, pharmacological, and non-pharmacological approaches. Pharmacological management primarily involves NSAIDs, glucocorticoids, DMARDs, TNF-α inhibitors (11). While effective for symptom management, these treatments pose significant long-term risks and may yield inadequate responses in a proportion of patients (11, 12). Consequently, there is growing clinical interest in exploring safe and effective alternative therapies.

Acupuncture, an important intervention in traditional Chinese medicine, has been widely used as a complementary and alternative therapy for patients with AS (13). It is traditionally held that this therapy works by regulating qi and blood, unblocking the meridians, and thus alleviating inflammation and pain (14). Compared with conventional pharmacotherapy, acupuncture provides rapid pain relief and sustains therapeutic benefits over the long-term via systemic regulatory mechanisms (15, 16). Moreover, acupuncture is associated with a very low incidence of adverse events (17), making it an increasingly attractive subject of clinical research. Research has demonstrated that acupuncture alleviates symptoms and improves function by regulating the neuro-immune-endocrine network to suppress inflammatory responses (18). According to some experimental and clinical studies, acupuncture has the potential to improve AS-related functional scores, such as BASDAI and BASFI, on modulating inflammatory factors such as IL-6, TNF-α, and IL-17 (19). Many studies (20) have shown that acupuncture and moxibustion have significant effects in alleviating pain symptoms, improving spinal mobility and improving patients’ quality of life. Another study (21, 22) has found that acupuncture can alleviate the inflammatory response of ankylosing spondylitis, thereby delaying the progression of the disease.

Although there is some evidence to support that acupuncture is effective in the treatment of AS, there is a lack of systematic and quantitative evidence-based evaluations. Moreover, existing systematic reviews predominantly assess short-term clinical symptom relief (23) and rarely examine long-term outcomes or the modulation of inflammatory cytokine profiles in AS patients receiving acupuncture therapy. Therefore, we conducted a systematic review and meta-analysis of RCTs to assess acupuncture’s impact on clinical symptoms and inflammatory biomarkers in AS, aiming to consolidate the evidence base for its management.

## 2 Methods

To assess the association of acupuncture therapy combinations with symptoms and immune markers in AS, we followed the Preferred Reporting Items for Systematic Evaluation and Meta-Analysis (PRISMA) statement (24). The evaluation program is registered in the PROSPERO International Prospective Systematic Evaluation Registry (CRD420251013146).

### 2.1 Literature search

The language of the search was limited to English or Chinese, and the databases PubMed, Embase, Cochrane Library, SinoMed, CNKI, Wanfang Data, and VIP were systematically searched in Chinese and English from the time of database construction to December 2024. The search terms included acupuncture, “Acupuncture,” “Electroacupuncture,” “Acupotomy,” “Acupoint,” “Ankylosis,” and “Acupuncture,” “Randomized controlled trial” “Ankylosing spondylitis.” The search strategy was adapted to the format of each database. We found that there are relatively few studies on acupuncture treatment for ankylosing spondylitis in international databases, so literature in international databases during preretrieval, we did not set search terms for literature types. And we adopted manual screening to avoid missing some papers that meet the criteria. The specific search strategy is presented in Supplementary Material 1.

### 2.2 Inclusion and exclusion criteria

Inclusion criteria: (1) the study was a randomized controlled trial (RCT). (2) The research subjects meet the New York criteria revised in 1984 (25) and the diagnostic criteria for axial spondyloarthritis proposed by The Assessment of SpondylArthritis international Society in 2009 (26). (3) The control group can be blank control, adjuvant therapy, conventional therapy, etc. (excluding acupuncture and related therapies). (4) Reporting of at least one outcome metric (27), the primary endpoint: VAS, CRP, ESR and time to morning stiffness; the secondary endpoints: IL-1, IL-6, IL-17, TNF-α, IgA, BASDAI, BASFI, and efficacy. Inclusion in the study must include either CRP or ESR.

Exclusion criteria: (1) Studies of patients with non-diagnosis or patients with certain diseases; (2) Non-RCT studies, such as observational studies, case reports, reviews, and conference reports; (3) Interventions other than acupuncture, or acupuncture combined with drugs, such as, new trials of the original drug (the clinical basis of the drug is not excluded); (4) Incomplete data or those who could not obtain the original data; (5) Repeated publication or multiple reports of the same study.

### 2.3 Literature screening and data extraction

Literature screening was independently conducted by two researchers using EndNote X9 software. The researchers first read the abstract and title of the paper to exclude duplicates, literature types, disease types, and research types that do not match, and then read the full text. According to the inclusion and exclusion criteria, the intervention methods containing acupuncture in the control group were excluded, and then literature with corresponding outcome indicators were screened to determine the final studies included. Two researchers independently extracted data from the included randomized controlled trials using standardized tables in Excel 2021. The extracted information includes first author, publication year, sample size, intervention, control, treatment duration, key outcome measures and their mean ± standard deviation (mean ± SD). The third experienced researcher made a ruling on any disagreements in the above process.

### 2.4 Assessment of risk of bias and reporting quality

The risk of bias of RCTs was independently assessed using the Cochrane Risk of Bias Assessment Tool version 2.0 (28); RoB 2 was applied to assess bias in six domains: random sequence generation, allocation concealment, blinding of participants and personnel, blinding of outcome assessment, incomplete outcome data, and selective reporting. For each of these areas, we rated studies as being at ‘high’, ‘low’ or ‘unclear’ risk of bias. A risk of bias map was generated. Two investigators were evaluated independently, and a third, more experienced investigator adjudicated any disagreements.

The reporting quality of the included RCTs was evaluated using the STRICTA checklist. STRICTA comprises six core domains: acupuncture rationale, details of needling, treatment regimen, other components of treatment, practitioner background, and control or comparator interventions. It further includes 17 sub-items designed to capture detailed information such as the selected acupuncture points, number of inserted needles, depths of insertion, response sought, needle stimulation, needle retention time, needle type, practitioner qualifications, training time, length of clinical experience and expertise. According to the STRICTA methodology, an item is marked as “yes” if it is fully reported; otherwise, it is recorded as “no.”

### 2.5 Statistical analysis

The meta-analysis was performed in Stata 15.0 software. Due to the uncertainties in acupuncture research (for example, in terms of research design, there are multiple and inconsistent internal parameters), the study internally employed a random-effects model. For continuous data, when the evaluation scale and units are unified (such as VAS, BASDAI, and BASFI), MD is used as the effect value, otherwise SMD is used as the effect value (such as IL-1, 1L-6, TNF-α). The effect value for dichotomous variables was the relative risk (RR). Heterogeneity was assessed using the I^2^ statistic. To evaluate the robustness of findings, sensitivity analyses were conducted by sequentially excluding individual studies. Publication bias was assessed for primary outcomes through visual inspection of funnel plots, supplemented by Begg’s and Egger’s tests. If statistical significance was indicated (*p* < 0.05), a trim-and-fill analysis was performed to evaluate the impact of potential bias. For outcomes with substantial heterogeneity (*I*^2^ > 50% and ≥10 studies), subgroup analyses were performed by intervention modality, treatment duration, and control type to explore potential sources.

### 2.6 GRADE approach

Moreover, we use the Grading of Recommendation, Assessment, Development and Evaluation (GRADE) approach to evaluate the reliability of the outcomes of the meta-analysis for each indicator (29). The GRADE framework systematically assesses the certainty of evidence in meta-analyses. It classifies evidence for each outcome into four levels (high to very low), starting from a baseline (high for RCTs, low for observational studies) and then adjusting for factors like risk of bias, inconsistency, and imprecision. This transparent process is vital for reliably informing clinical and policy decisions.

## 3 Results

### 3.1 Literature screening process and study characteristics

**Figure 1:**
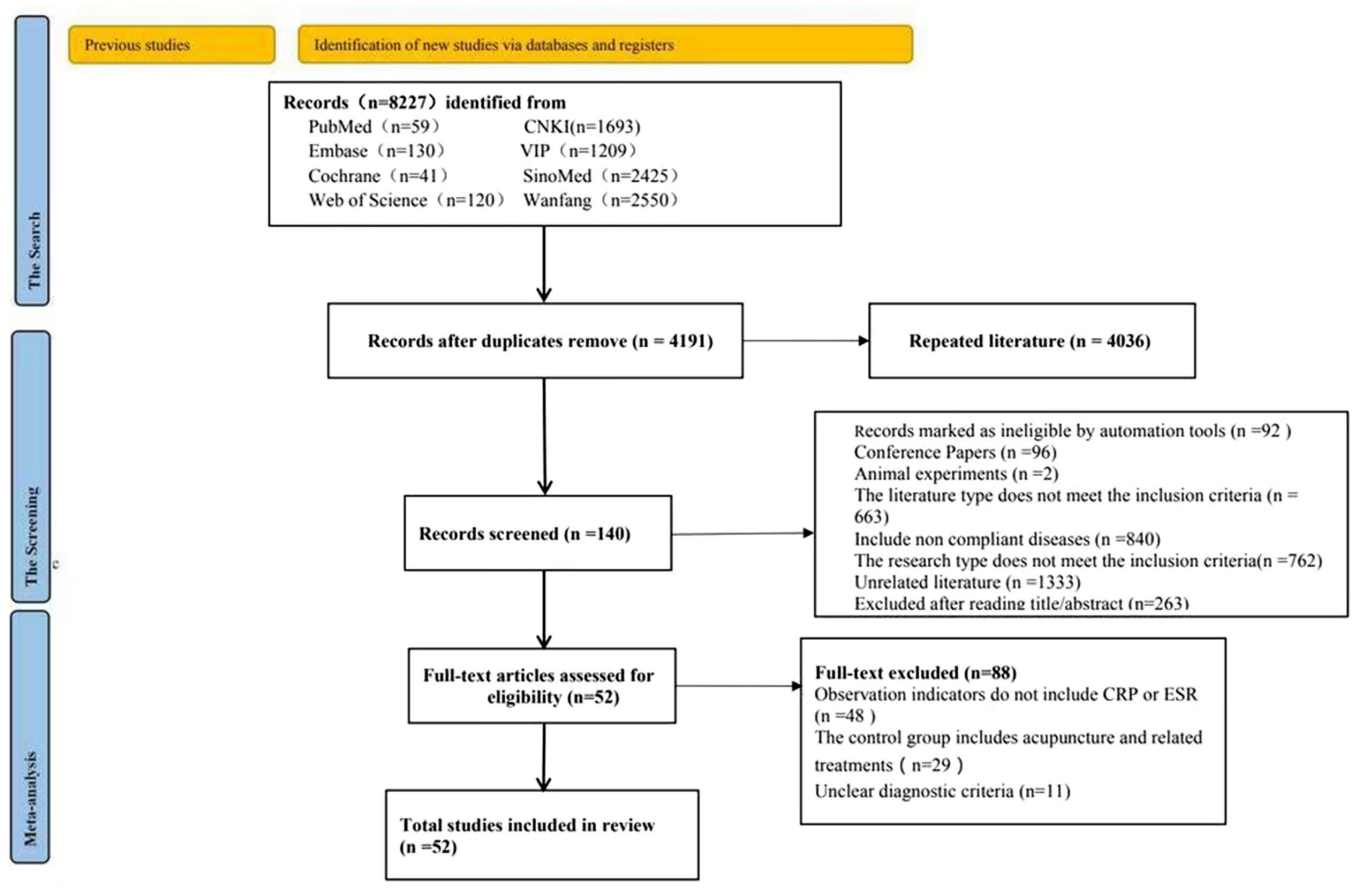
PRISMA 2020 flow diagram for updated systematic reviews, which included searches of databases, registers, and other sources.

The initial literature search generated 8,227 records. After removing duplicate items, there are still 4,191 records that need to be filtered. Select titles and abstracts, literature types that do not match, conference papers, disease types that do not match, etc. 4,051 studies were excluded, and the remaining 140 were included in full-text screening. After detailed full text screening, literature that did not meet the inclusion criteria, such as mismatched intervention measures, mismatched outcome indicators, unclear diagnostic criteria, etc., were excluded. This review included 52 randomized controlled trials. All studies compared the effects of acupuncture and acupuncture combined with medication on patients with ankylosing spondylitis. The study selection process is summarized in the PRISMA flow diagram (Figure 1 and Table 1).

**Table 1:** Research characteristics included in RCTs.

Studies (year)	No. of participants (T/C)	Intervention of treatment course of disease (y, years; m, months; d, days)	Intervention of control course of disease (y, years; m, months; d, days)	Specific acupoints or treatment areas	Outcome
Bai et al. (2014) (30)	60/60T: Mean age (32.5 ± 6.7) y; C: Mean age (34.2 ± 7.1) y	Acupuncture (25 min) + Conventional therapy (Sulfasalazine [0.75 g, tid] + Diclofenac [75 mg, qd]) 12w	Conventional therapy (Sulfasalazine [0.75 g, tid] Diclofenac [75 mg, qd]) 12w	EX-B2, UB23, UB25, UB40 UB54, GB30	VAS, ESR, CRP
Chen (2009) (55)	c	Acupuncture (30 min, qd) 4w	Functional training 4w	EX-B2, DU2, DU3, DU4, DU14, DU8, UB23, UB31, UB32, UB33, UB34, Du Meridian	ESR, CRP, Morning stiffness, Clinical efficacy
Chen et al. (2018) (31)	30/30 T: Mean age (25.21 ± 3.11) y, Mean duration (2.76 ± 1.56) y; C: Mean age (25.42 ± 3.82) y, Mean duration (2.39 ± 1.78) a	Acupuncture (Once every 4 weeks) + Conventional therapy (Sulfasalazine [0.5 g, tid]) 12w	Conventional therapy (Sulfasalazine [0.5 g, tid]) 12w	Sacroiliac joint area	VAS, ESR, CRP, BASDAI, BASFI
Gui-yi et al. (2021) (32)	64/64 T: Mean age (34.4 ± 5.2) y, Mean duration (15.8 ± 3.8) m; C: Mean age (33.5 ± 5.3) y, Mean duration (15.2 ± 3.9) m	Acupuncture (30 min, qd, 5 times every week) Conventional therapy (Sulfasalazine [1 g, tid] + Meloxicam [7.5 mg, qd]) 8w	Conventional therapy (Sulfasalazine [1 g, tid] + Meloxicam [7.5 mg, qd]) 8w	EX-B2, SI3, DU3, DU4, DU6, DU8, DU9, DU14	VAS, ESR, CRP, BASDAI, BASFI, Morning stiffness, Clinical efficacy
Dong (2017) (33)	30/30T: Mean age (51 ± 1) y, Mean duration (5.82 ± 1.20) y; C: Mean age (48 ± 2) y; Mean duration (5.41 ± 1.21) y	Acupuncture (25 min, qw) 8w	Conventional therapy (Methotrexate [10 mg, qw] + Sulfasalazine [0.25 g, bid]) 8w	L2-S1 lamina (attachment of deep muscles in the waist), posterior one-third of the iliac spine, and inner edge of the posterior superior iliac spine	VAS, IL-1, IL-6, Morning stiffness, Clinical efficacy
Gao (2019) (34)	20/20T: Mean age (28.2 ± 7.8) y; C: Mean age (30.1 ± 5.8) y	Acupuncture (30 min, qd) + Conventional therapy (Sulfasalazine [Week 1: 0.5 g, bid; Week 2: 0.5 g,bid; Week 3:1 g, bid]) 4w	Conventional therapy (Sulfasalazine [Week 1: 0.5 g, bid; Week 2:0.5 g, bid; Week 3: 1 g, bid]) 4w	EX-B2	ESR, CRP, B ASDAI, BASFI, Morning stiffness, Clinical efficacy
Guo (2021) (62)	36/36T: Mean age (39.67 ± 8.23) y, Mean duration (42.67 ± 11.18) m; C: Mean age (38.74 ± 1.45) y, Mean duration (43.58 ± 11.34) m	Acupuncture (3–5 min, qod) + Conventional therapy (Detoxification and Enhancement Oral Liquid [30 mL, bid]) 6.4w	Conventional therapy (Sulfasalazine [Week 1: 0.5 g, bid; Week 2–12: 1 g, bid]) 12w	EX-B2, SI3, UB65	ESR, CRP, Morning stiffness, Clinical efficacy
Guo (2024) (35)	47/47 T: Mean age (31 ± 8) y, Mean duration (8.22 ± 3.21) y; C: Mean age (32 ± 8) y, Mean duration (8.48 ± 3.35) y	Acupuncture (30 min, qd, Five times a week) + Conventional therapy (Intravenous infusion of Infliximab [Each dose is 5 mg/kg, Take medication once in weeks 2and 6, once every 6 weeks]) 32w	Conventional therapy (Intravenous infusion of Infliximab [Each dose is 5 mg/kg, Take medication once in weeks 2and 6,once every 6 weeks]) 32w	UB62, KID6	VAS, ESR, CRP, BASDAI, BASFI, Clinical efficacy
Hou (2019) (36)	30/32T: Mean age (43.96 ± 7.57) y, Mean duration (7.75 ± 2.95) y; C: Mean age (41.28 ± 9.33) y, Mean duration (7.03 ± 2.67) y	Acupuncture (25 min) 4w	Conventional therapy (Celecoxib) 4w	DU20, DU14, DU3, DU4, SI3, UB26, UB25, KID3, UB60	VAS, ESR, CRP, Clinical efficacy
Jin (2021) (37)	25/25T: Mean age (28.36 ± 8.52) y, Mean duration (7.36 ± 2.41) y, C: Mean age (27.42 ± 6.52) y, Mean duration (7.72 ± 1.52) y	Acupuncture (6 h qd) + Conventional therapy (Celecoxib [0.2 g, bid]) 2w	Conventional therapy (Celecoxib [0.2 g, bid]) 2w	EX-B2, DU14	VAS, ESR, CRP, BASDAI, BASFI, Clinical efficacy
Kang (2021) (38)	45/45T: Mean age (33.21 ± 4.19) y, Mean duration (8.25 ± 3.32) y; C: Mean age (33.54 ± 4.26) y, Mean duration (7.75 ± 3.21) y	Acupuncture (30 min, qd) + Conventional therapy (Leflunomide [20 mg, qd] + Meloxicam [75 g, qd] + Sulfasalazine [0.25 g, bid]) 12w	Conventional therapy (Leflunomide [20 mg, qd] + Meloxicam [75 g, qd] + Sulfasalazine [0.25 g, bid]) 12w	EX-B2, DU1, DU2, DU5, DU7, DU9, DU10, DU14, DU20	VAS, IL-17, BASDAI, BASFI, Clinical efficacy
Li (2015) (63)	33/31	Acupuncture (5–20 min, biw) 12w	Conventional therapy (Sulfasalazine [Week 1:0.25 g, bid; Week 2:0.5 g, bid; Week 3:0.75 g, bid, Week 4–12:1 g, bid]) 12w	Ashi acupoint, UB23, DU14, UB20, UB31, UB32, UB33, UB34	ESR, CRP, Clinical efficacy
Li (2016) (39)	40/42	Acupuncture (24 h, qod. Depending on the patient’s condition gradually extend to once a week) + Conventional therapy (Meloxicam [15 mg, qn] + Sulfasalazine [0.75 g, bid]) 12w	Conventional therapy (Meloxicam [15 mg, qn] + Sulfasalazine [0.75 g, bid]) 12w	Search for myofascial trigger points or trigger points (MTrP) in the waist and back	VAS, ESR, CRP, TNF-α, IL-6
Li (2020) (64)	128/127T: Mean age (41.67 ± 9.48) y, Mean duration (5.99 ± 3.07) y; C: Mean age (40.25 ± 10.59) y, Mean duration (6.49 ± 2.73) y	Acupuncture (1–3 times every week, 4w) + Conventional therapy (Sulfasalazine [Week 1:0.25 g, tid; Week 2:0.5 g, tid; Week 3:0.75 g, tid; Week 4–12:1 g, tid]) 12w	Conventional therapy (Meloxicam [7.5 mg, qd] + Sulfasalazine [Week 1: 0.25 g, tid; Week 2: 0.5 g, tid; Week 3: 0.75 g, tid; Week 4–12: 1 g, tid]) 12w	Select joint capsules and surrounding tissues of diseased hip, knee, shoulder, elbow, ankle, sacroiliac and other joints as treatment points	CRP, TNF-α, IL-6, Clinical efficacy
Li (2021) (40)	52/52T: Mean age (27.14 ± 2.61) y, Mean duration (1.24 ± 0.31) y; C: Mean age (26.92 ± 2.57) y, Mean duration (1.31 ± 0.34) y	Acupuncture (25 min) + Conventional therapy (Loxoprofen sodium [60 mg, tid] + Sulfasalazine [1 g, bid]) 8w	Conventional therapy (Loxoprofen sodium [60 mg, tid] + Sulfasalazine [1 g, bid]) 8w	DU2, DU3, DU4, DU5, DU6, DU7, DU8, DU9, DU10, DU11, DU11, DU13, DU14, DU20	VAS, IL-6, IL-17, TNF-α, Morning stiffness, Clinical efficacy
Liang (2014) (41)	43/44T: Mean age (40 ± 10) y, Mean duration (43.2 ± 10.3) m; C: Mean age (42 ± 10) y, Mean duration (45.3 ± 9.8) m	Acupuncture (30 min, qd, 5 consecutive times every week) + Conventional therapy (Sulfasalazine [Day 1 to Day 3:0.25 g, tid; Days 4 to 28: 0.5 g, tid]) 4w	Conventional therapy (Sulfasalazine [Day 1 to Day 3:0.25 g, tid; Days 4 to 28: 0.5 g, tid]) 4w	EX-B2, UB31, UB32, UB33, UB34	VAS, ESR, CRP, Morning stiffness
Ling (2014) (42)	37/37T: Mean age (24.81 ± 1 2.90) y, Mean duration (3.93 ± 7.08) y; C: Mean age (25.67 ± 12.43) y, Mean duration (4.24 ± 7.66) y	Acupuncture (qw) + Conventional therapy (Sulfasalazine [0.5 g, tid]) 12w	Conventional therapy (Sulfasalazine [0.5 g, tid]) 12w	EX-B2	VAS, ESR, CRP, TNF-α
Liu (2019) (56)	40/40T: Mean age (36.0 ± 5.4) y, Mean duration (3.0 ± 1.2) y; C: Mean age (36.5 ± 5.2) y, Mean duration (3.0 ± 1.0) y	Acupuncture (30 min) + Conventional therapy (Diclofenac [75 mg, qd] + Sulfasalazine [Week 1:1 g, qd; Week 2–8: 2 g, qd]) 8w	Conventional therapy (Diclofenac [75 mg, qd] + Sulfasalazine [Week 1: 1 g, qd; Week 2–8: 2 g, qd]) 8w	EX-B2, Ashi acupoint, DU14, UB23, UB25, UB40, UB54, GB30	CRP, TNF-α, BASDAI, BASFI,
Lu (2024) (65)	34/34T: Mean age (27.93 ± 5.70) y, Mean duration (3.68 ± 1.20) y; C: Mean age (27.27 ± 5.9) y, Mean duration (3.75 ± 1.10) y	Acupuncture (4 h, qod) + Conventional therapy (Celecoxib [0.2 g, qd] + Sulfasalazine [0.25 g, bid]) 8w	Conventional therapy (Celecoxib [0.2 g, qd] + Sulfasalazine [0.25 g, bid]) 8w	Myofascial trigger point (MTrP) or affected muscle (sacroiliac joint, lumbar back, neck, etc.)	ESR, CRP, TNF-α, BASDAI, BASFI
Luo (2024) (66)	30/30T: Mean age (39.67 ± 2.32) y, Mean duration (8.50 ± 1.45) y; C: Mean age (43.47 ± 2.39) y, Mean duration (8.83 ± 1.16) y	Acupuncture (30 min, three times every week) + Conventional therapy (Celecoxib [0.2 g, qd] + Sulfasalazine [0.5 g, bid]) 4w	Conventional therapy (Celecoxib [0.2 g, qd] + Sulfasalazine [0.5 g, bid]) 4w	DU14, DU20, HT7, UB18, UB23, DU3, GB34, SP6	CRP, BASDAI, BASFI, Clinical efficacy
Meng (2022) (67)	40/40T: Mean age (25.11 ± 1.25) y, Mean duration (8.11 ± 2.52) y; C: Mean age (24.50 ± 1.23) y, Mean duration (7.05 ± 2.67) y	Acupuncture (40 min, qd) + Conventional therapy (Thalidomide [50 mg, tid]) 12w	Conventional therapy (Thalidomide [50 mg, tid]) 12w	Ashi acupoint, EX-B2, UB18, UB23, GB20	ESR, CRP, TNF-α, IL-6, BASDAI, Clinical efficacy
Ruan (2010) (43)	30/27	Acupuncture (Twice every 4 weeks)8w + Conventional therapy (Sulfasalazine [Week 1: 0.25 g, tid; Week 2: 0.5 g, tid; Week 3: 0.75 g, tid. 24w] + Diclofenac [75 mg, bid]) 12w	Conventional therapy (Sulfasalazine [Week 1: 0.25 g, tid; Week 2: 0.5 g, tid; Week 3: 0.75 g, tid. 24w] + Diclofenac [75 mg, bid]) 12w	Select the layer with more severe sacroiliac joint damage	VAS, ESR, CRP, BASDAI, BASFI, Clinical efficacy
She (2019) (68)	68/34T: Mean age (32.38 ± 7.87) y, Mean duration (4.57 ± 2.84) y; C: Mean age (32.21 ± 7.98) y, Mean duration (5.08 ± 3.01) y	Acupuncture (qd) 12w	Conventional therapy (Celecoxib [0.1 g, bid]) 12w	Du Mai acupoint, Foot Sun Bladder Meridian acupoint, Ashi acupoint, EX-B2, DU14, LI11, SP9, REN4, UB23, UB25	ESR, CRP, BASDAI, BASFI, Clinical efficacy
Sun (2015) (44)	24/25	Acupuncture (qd, five times every week) 4w	Conventional therapy (Sulfasalazine [Week 1: 0.25 g, tid; Week 2: 0.5 g, tid; Week 3–4:0.75 g, tid] + Naproxen [0.2 g, bid] + Tripterygium [15 mg, tid]) 4w	EX-B2, Ashi acupoint, the Foot Sun Bladder Meridian and Du Mai Meridian on the Back and Waist	VAS, ESR, CRP, BASDAI, BASFI, Morning stiffness
Tian (2011) (45)	43/40T: Mean age (36.17 ± 8.97) y, Mean duration (5 ± 2.41) y; C: Mean age (35.19 ± 9.31) y, Mean duration (5 ± 2.50) y	Acupuncture (qw) 8w	Conventional therapy (Sulfasalazine [Week 1: 0.25 g, tid; Week 2: 0.5 g, tid; Week 3–8:0.75 g, tid]) 8w	EX-B2, UB11, UB23, GB34, ST36, UB40	VAS, ESR, CRP, Morning stiffness, Clinical efficacy
Wang (2010) (69)	30/30	Acupuncture (week 1–week 4: once every 3 days, week 5–week 12: once every 4 weeks) 12w + Conventional therapy (Thalidomide [50 mg, qd; Add 50 mg every 10 days maintain at 150 mg for 24 weeks] or Sulfasalazine [0.25 g, bid; Add 0.25 g per week to 1.0 g, 24w])	Conventional therapy (Nimesulide [0.1 g, bid] 12w + Thalidomide [50 mg, qd; Add 50 mg every 10 days maintain at 150 mg for 24 weeks] or Sulfasalazine [0.25 g, bid; Add 0.25 g per week to 1.0 g, 24w])	Select areas with tenderness and muscle contraction on both sides of the spine	VAS, ESR, CRP
Wang (2015) (70)	25/25	Acupuncture (20 min) 2-week interval, determine whether to continue treatment based on changes in the patient’s condition	Conventional therapy (Sulfasalazine [0.75 mg, tid] + Meloxicam [15 mg, qd]) 6w	Tender points in the affected area	VAS, ESR, CRP, BASDAI, Clinical efficacy
Wang (2017) (80)	38/36T: Mean age (29 ± 5) y, C: Mean age (30 ± 5) y	Acupuncture (5 h, qod, According to the patient’s condition, gradually extend to once a week) 24w	Conventional therapy (Celecoxib [0.2 g, qd] + Sulfasalazine [Week 1: 0.25 g, tid; Week 2: 0.5 g, tid; Week 3: 0.75 g, tid, Week 4–24: 1 g, bid] + Methotrexate [Week 1: 5 mg, qw; Week 2:7.5 mg, qw; Week 3–24: 10 mg, qw]) 24w	Select the tight, linear, and nodular muscles of the waist, back, and buttocks	TNF-α, IL-1, IL-6, BASDAI, BASFI
Wang (2022) (46)	30/30T: Mean age (36.13 ± 8.15) y, Mean duration (6.33 ± 3.43) y; C: Mean age (38.37 ± 7.53) y, Mean duration (6.83 ± 3.48) y	Acupuncture (60–70 min, biw) + Conventional therapy (Sulfasalazine [Week 1: 0.25 g, tid; Week 2: 0.5 g, tid; Week 3: 0.75 g, tid; Week 4–8: 1 g, bid]) 8w	Conventional therapy (Sulfasalazine [Week 1: 0.25 g, tid; Week 2: 0.5 g, tid; Week 3: 0.75 g, tid; Week 4–8: 1 g, bid]) 8w	SP3, SJ6	VAS, ESR, CRP, BASDAI, BASFI, Clinical efficacy
Wang (2022) (71)	45/45T: Mean duration (10.80 ± 6.19) y; C: Mean duration (10.71 ± 6.34) y	Acupuncture (30 min, qd, 5 times a week) + Conventional therapy (Sulfasalazine [Week 1: 2 pieces, qd; Week 2: 2 pieces, bid, Week 3–8: 3 pieces, bid]) 8w	Conventional therapy (Sulfasalazine [Week 1: 2 pieces, qd; Week 2: 2 pieces, bid, Week 3–8: 3 pieces, bid]) 8w	Foot sun meridian tendon lesion site	ESR, CRP, Clinical efficacy
Wang (2022) (78)	45/45T: Mean age (49.3 ± 3.4) y, Mean duration (5.38 ± 1.29) y; C: Mean age (48.6 ± 3.3) y, Mean duration (5.31 ± 1.26) y	Acupuncture (30 min, qd, Rest for 3 days every 10 days) 36d + Conventional therapy (Sulfasalazine [1 g, bid]) 30d	Conventional therapy (Sulfasalazine [1 g, bid]) 30d	Foot sun meridian tendon lesion site	ESR, BASDAI, BASFI, TNF-α, Clinical efficacy
Wang (2022) (47)	32/31T: Mean age (31.28 ± 3.15) y, Mean duration (10.92 ± 2.48) m; C: mean age (32.05 ± 3.21) y, Mean duration (11.13 ± 2.39) m	Acupuncture (qiw, Rest for 1 week after 2 weeks) + Conventional therapy (Sulfasalazine [0.5 g, tid]) 8w	Conventional therapy (Sulfasalazine [0.5 g, tid]) 8w	Pain points in the spinous processes of the lumbar spine, bilateral articular processes, transverse processes, abdomen, sacral spinous processes, and medial margin of the sacroiliac joint	VAS, ESR, CRP, TNF-α, BASDAI, BASFI, Clinical efficacy
Wang (2022) (48)	54/53T: Mean age (33 ± 5) y, Mean duration (4.71 ± 0.80); C: Mean age (32 ± 5) y, Mean duration (4.62 ± 0.74) y	Acupuncture (20 min, qod) + Auricular point sticking therapy (2 min, tid) 6w	Conventional therapy (Meloxicam [15 mg, qd]) 6w	EX-B2, DU2, DU6, DU12, UB23, GB34, UB16, DU5, DU8, DU9, DU14, DU3, Ashi acupoint	CRP, VAS, TNF-α, IL-17, Morning stiffness, Clinical efficacy
Wu (2009) (79)	40/40 T: Mean age (31.6 ± 11.3) y, Mean duration (5.3 ± 5.6); C: Mean age (21 ± 8) y, Mean duration (6 ± 5.5) y	Acupuncture (20–30 min, qd)3 m (Hospitalization for 1.5 months outpatient treatment continued for 3 months)	Conventional therapy (Diclofenac [50 mg, tid] + Sulfasalazine [Week 1: 0.5 g, tid; Starting from the second week: 1 g, tid])3 m (Hospitalization for 1.5 months outpatient treatment continued for 3 months)	EX-B2, UB23, UB31, UB32, UB33, UB34	ESR, Clinical efficacy
Wu (2018) (57)	30/30 T: Mean age (23.5 ± 2.2) y, Mean duration (5.0 ± 0.4) y; C: Mean age (23.4 ± 2.5) y, Mean duration (4.6 ± 0.3) y	Acupuncture (30 min, qd, 6 consecutive days of treatment and 1 day of rest) + Conventional therapy (Diclofenac [0.75 mg, qd] + Sulfasalazine [0.75 g, tid]) 12w	Conventional therapy (Diclofenac [0.75 mg, qd] + Sulfasalazine [0.75 g, tid]) 12w	Ashi acupoint, DU3, DU, REN3, REN4, REN6, REN7, REN9, REN10, REN12, SP15, KID13, ST24, ST26	ESR, CRP, IgA, Morning stiffness, Clinical efficacy
Wu (2023) (58)	39/39T: Mean age (40 ± 7) y, Mean duration (4.71 ± 2.82) y; C: Mean age (38 ± 5) y, Mean duration (5.21 ± 1.78) y	Acupuncture (20 min, qd) + Conventional therapy (Indomethacin [75 mg, qd] + Sulfasalazine [1 g, bid]) 3w	Conventional therapy (Indomethacin [75 mg, qd] + Sulfasalazine [1 g, bid]) 3w	UB62, KID6, UB23, UB25	CRP, BASFI, TNF-α, IL-17, Morning stiffness, Clinical efficacy
Xie (2009) (72)	11/15T: Mean age (40 ± 7) y; C: Mean age (38 ± 5) y	Acupuncture (Once every 15 days) 8w	Conventional therapy (Sulfasalazine [750 mg, bid] + Xilebao Capsules [200 mg, qd]) 8w	The waist is located along the edge of the pelvic iliac crest where the inner edge of the posterior superior iliac spine and one-third of the posterior iliac crest muscles attach, namely the muscle attachment area around the sacroiliac joint, as well as the vertebral plate adjacent to the L3-S2 spinous process; The buttocks are the attachment site of the gluteus medius muscle, the iliotibial bundle, the iliac wing, the upper edge of the ischial foramen and the intertrochanteric fossa of the femur, as well as the posterior inferior iliac spine, the outer edge of the sacroiliac joint, and the upper part of the ischial tuberosity	ESR, CRP, BASDAI, BASFI
Xing (2021) (59)	30/30T: Mean age (24.51 ± 3.10) y, Mean duration (4.80 ± 0.61); C: Mean age (25.00 ± 2.61) y, Mean duration (4.81 ± 0.30) y	Acupuncture (34–35 min, 6 times every week) + Conventional therapy (Celecoxib [0.2 g, qd] + Sulfasalazine [Week 1: 0.5 g, bid; Week 2: 0.75 g, bid; Week 3–12:1.0 g, bid]) 12w	Conventional therapy (Celecoxib [0.2 g, qd] + Sulfasalazine [Week 1: 0.5 g, bid; Week 2: 0.75 g, bid; Week 3–12:1.0 g, bid]) 12w	Ashi acupoint, EX-B2, DU2, DU4, UB25, GB30, ST31, UB40, GB34, REN3, REN4, REN6, REN7, REN9, REN10, REN12, SP15, KID13, ST24, ST26	ESR, CRP, IgA, Morning stiffness
Yang (2010) (60)	30/30T: Mean age (43.6 ± 12.5) y, Mean duration (9.5 ± 5.4) m; C: Mean age (42.5 ± 12.2) y, Mean duration (9.2 ± 5.2) m	Acupuncture (30 min, qd) 12w	Conventional therapy (Indomethacin [50 mg, tid] + Sulfasalazine [1 g, bid] + Methotrexate [Week 1: 2.5 mg; Week 2: 5 mg; Week 3:7.5 mg; Week 4: 10 mg, Maintain 10–15 mg per week]) 12w	EX-B2, DU14, GB20, UB23	CRP, Morning stiffness, Clinical efficacy
Yang (2015) (81)	40/40T: Mean age (31.90 ± 8.34) y, Mean duration (26.65 ± 4.07) m; C: Mean age (34.10 ± 5.19) y, Mean duration (24.06 ± 4.64) m	Acupuncture (Week 1–4:qw; Week 5–12:biw) + Conventional therapy (Diclofenac [50 mg, bid]) 12w	Conventional therapy (Sulfasalazine [Week 1: 0.25 g, bid; Week 2: 0.5 g, bid; Week 3–12: 1 g, bid] + Diclofenac [50 mg, bid]) 12w	① Sacral joint: Select 1/2 parts above the sacral joint, the lower edge of the spinous process of the fifth lumbar vertebra vertically downward by 3 cm, and then parallel outward by 3 cm; ② Soft tissue around the spine: Select the site of spinal stiffness, pain, and discomfort	IL-6, TNF-α BASDAI, BASFI
Yang (2020) (49)	34/34T: Mean age (42.85 ± 8.56) y, Mean duration (6.10 ± 3.03) m; C: Mean age (42.76 ± 9.69) y, Mean duration (6.08 ± 3.04) m	Acupuncture (20 min, 3–4 times every week) 8w	Conventional therapy (Sulfasalazine [1 g, bid] + Imrecoxib [0.1 g, bid]) 8w	EX-B2, UB11, UB23, DU14, GB20	VAS, ESR, CRP, Clinical efficacy
You (2016) (50)	45/45T: Mean age (25.10 ± 3.32) y, Mean duration (6.10 ± 3.03) m; C: Mean age (25.30 ± 3.94) y, Mean duration (6.08 ± 3.04) m	Acupuncture (Once every other month) 24w	Conventional medication 24w	Sacroiliac joint area	ESR, CRP, BASDAI, BASFI
Yuan (2022) (61)	30/30T: Mean age (35.13 ± 2.08) y, Mean duration (37.32 ± 1.28) m; C: Mean age (35.26 ± 2.14) y, Mean duration (37.28 ± 1.36) m	Acupuncture (30 min, 3 times a week once every other day) 12w	Conventional therapy (Meloxicam [7.5 mg, bid] + Sulfasalazine [Week 1:0.25 g, tid; Week 2:0.5 g, tid; Week 3–12:0.75 g, tid]) 12w	DU14, DU20, DU3, DU4, KID3, UB52, UB60	ESR, CRP, Morning stiffness, Clinical efficacy
Zhan (2019) (73)	24/24T: Mean age (28.3 ± 5.2) y, Mean duration (2.69 ± 1.72) y; C: Mean age (28.5 ± 6.3) y, Mean duration (2.88 ± 1.53) m	Acupuncture (20 min, qw) 4w	Conventional therapy (Sulfasalazine [Week 1: 0.5 g, qd; Week 2: 0.5, bid; Week 3–4: 0.75 g, bid]) 4w	Sacroiliac joint capsule, L1-S1 facet joint capsule, thoracic facet joint capsule, C2-C7 facet joint capsule	ESR, CRP, IL-6, TNF-α, Clinical efficacy
Zhang (2010) (74)	43/43	Acupuncture (30 min, qd, once every other day) + Conventional therapy (Sulfasalazine [0.5, tid] + Meloxicam [7.5 mg, qd] + Methotrexate [7.5 mg, qw]) 12w	Conventional therapy (Sulfasalazine [0.5, tid] + Meloxicam [7.5 mg, qd] + Methotrexate [7.5 mg, qw]) 12w	EX-B2, DU2, DU3, DU4, DU14, Ashi acupoint	ESR, CRP, TNF-α
Zhang (2019) (51)	45/45T: Mean duration (5.09 ± 1.91) y; C: Mean duration (5.14 ± 2.21) y	Acupuncture (30 min, qd) + Conventional therapy (Sulfasalazine [1 g, bid] + Meloxicam [7.5 mg, qd]) 8w	Conventional therapy (Sulfasalazine [1 g, bid] + Meloxicam [7.5 mg, qd]) 8w	EX-B2	VAS, ESR, Clinical efficacy
Zhao (2016) (52)	31/31	Acupuncture (30 min, qd, After continuous treatment for 5 days rest for 2 days) + Conventional therapy (Sulfasalazine [0.5 g, bid] + Etoricoxib [60 mg, qd]) 4w	Conventional therapy (Sulfasalazine [0.5 g, bid] + Etoricoxib [60 mg, qd]) 4w	EX-B2, Pain area at tendon attachment point	VAS, ESR, CRP, BASDAI, BASFI, Morning stiffness, Clinical efficacy
Zhao (2023) (53)	29/28T: Mean age (34.97 ± 11.31) y; C: Mean age (33.33 ± 10.70) y	Acupuncture (30 min, qd, Treat once every other day) + Conventional therapy (Celecoxib [0.2 g, bid]) 4w	Conventional therapy (Celecoxib [0.2 g, bid]) 4w	TSBh1-8, 9, 10, RFh1–6, RFh2-6, DXh2-6, 8, 11, DWSg, DNsz, DZBh1-5	VAS, ESR, CRP, BASDAI, Clinical efficacy
Zhao (2024) (54)	30/30T: Mean age (35.2 ± 2.2) y, Mean duration (306 ± 1.13) y; C: Mean age (33.7 ± 1.9) y, Mean duration (3.20 ± 1.26) y	Acupuncture (30 min, qd. Treat 5 times every week) 4w	Conventional medication 4w	EX-B2	VAS, ESR, CRP, IgA, BASFI, Morning stiffness, Clinical efficacy
Zheng (2015) (75)	30/30T: Mean age (24.77 ± 5.41) y, Mean duration (1.3 ± 2.1) y; C: Mean age (25.48 ± 6.33) y, Mean duration (1.3 ± 2.1) y	Acupuncture (10 min, Once every other day) 12w	Conventional therapy (Diclofenac [75 mg, qd] + Sulfasalazine [Week 1: 0.25 g, tid; Week 2: 0.5 g, tid; Week 3: 0.75 g, tid]) 12w	The strongest point of thermal sensitivity	ESR, CRP, BASDAI, BASFI, Clinical efficacy
Zheng (2020) (76)	44/44T: Mean age (29.67 ± 9.35) y, Mean duration (6.35 ± 3.27) y; C: Mean age (30.35 ± 9.26) y, Mean duration (6.72 ± 3.44) y	Acupuncture (30 min, Once every other day) + Conventional therapy (Sulfasalazine [weeks: 1–2: 0.25 g, tid; weeks 3–12: 0.5 g, tid]) 12w	Conventional therapy (Sulfasalazine [Week 1–2: 0.25 g, tid; Week 3–12: 0.5 g, tid]) 12w	EX-B2, Du Mai acupoint	ESR, CRP, BASDAI, BASFI, Morning stiffness, Clinical efficacy
Zhou (2011) (77)	28/28T: Mean age (27.31 ± 9.25) y; C: Mean age (25.74 ± 10.12) y	Acupuncture (30 min, qd) + Conventional therapy (Celecoxib [0.2 g qd] + Sulfasalazine [1.0 g, bid]) 22d	Conventional therapy (Celecoxib [0.2 g qd] + Sulfasalazine [1.0 g, bid])	EX-B2, DU14, UB11, UB18, UB23, Bai Lao acupoint	ESR, CRP, BASDAI, BASFI

### 3.2 Results of risk of bias

Among the 52 included trials, 32 were judged to have a moderate risk of bias, whereas the remaining 20 were rated as low risk.

Analysis indicates that the standardization of randomization procedures, the effectiveness of allocation concealment, and the completeness of blinding implementation represent the primary sources of bias. All studies exhibited design flaws in at least one aspect: either selection bias (e.g., lack of allocation concealment) or implementation bias (failure to blind subjects and researchers), highlighting methodological rigor deficiencies in some studies.

**Figure 2:**
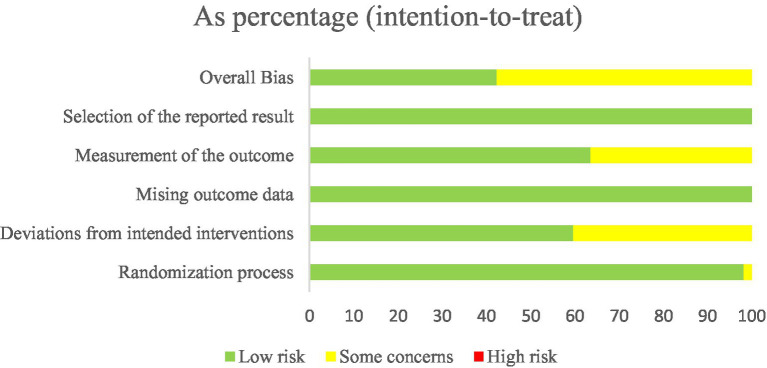
Overall distribution of bias risk.

**Figure 3:**
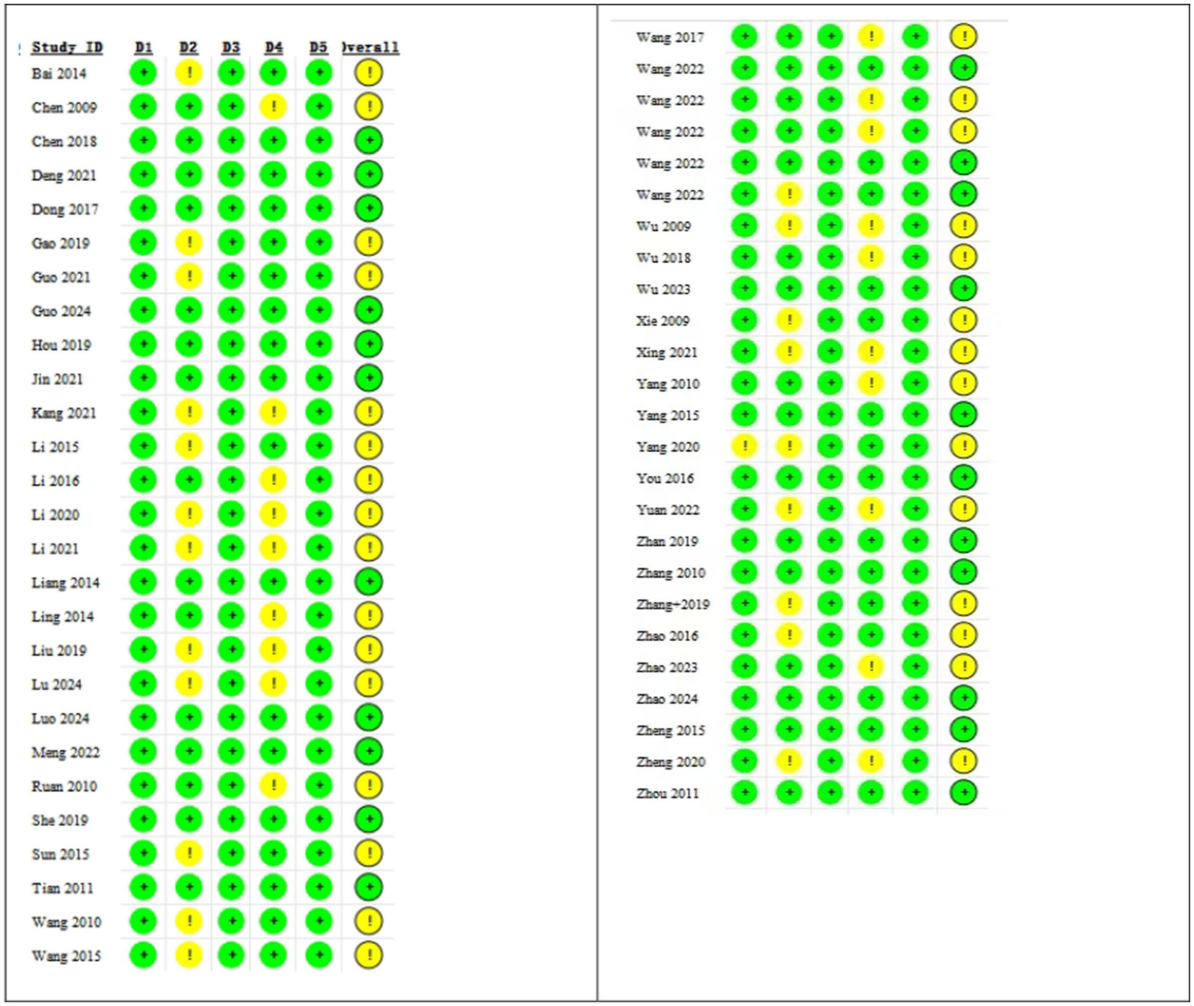
Detailed deviation risk assessment.

The overall distribution of bias risk is presented in Figure 2. Figure 3 offers a detailed bias risk assessment for each study outcome, providing visual validation for conclusion reliability.

**Figure 4:**
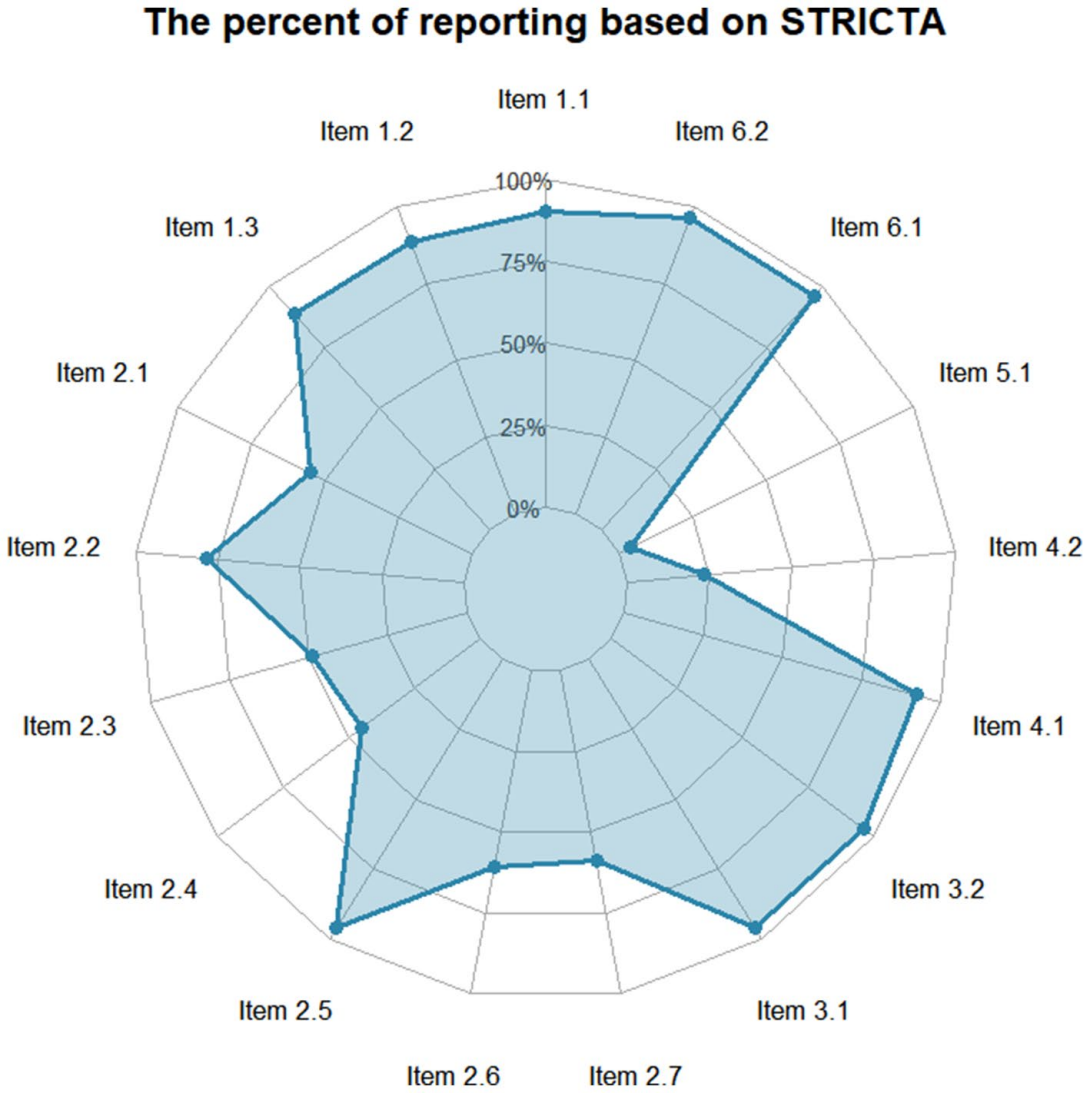
Radar chart.

Figure 4 display the percentage of reporting based on the STRICTA guidelines. The reporting rates varied across the items, which were categorized into high (>80%), moderate (50–80%), and low (<50%) reporting groups.

Most items related to the acupuncture rationale, treatment regimen, and comparator interventions were reported with high frequency. Specifically, Item 2.5 (needle stimulation), Item 3.1 and 3.2 (number, frequency and duration of sessions), and Item 6.1 and 6.2 (comparator interventions) achieved the highest reporting rate (50/52, 96.15%). These were followed by Item 4.1 (details of other interventions) (48/52, 92.31%) and Item 1.1 (style of acupuncture) (47/52, 90.38%). Additionally, Item 1.2 (reasoning for treatment) and Item 1.3 (extent of variation) were both reported in 88.46% (46/52) of the studies.

Items regarding the details of needling generally showed moderate reporting rates. Item 2.2 (names of points) was reported in 78.85% (41/52) of the studies, while Item 2.6 (needle retention) and Item 2.7 (needle type) were reported in 61.54% (32/52) and 59.62% (31/52), respectively. Item 2.1 (number of needle insertions) and Item 2.3 (depth of insertion) appeared in 55.77% (29/52) and 50.00% (26/52) of the reports.

Conversely, three items demonstrated low reporting rates (less than 50%). Item 2.4 (response sought) was reported in 46.15% (24/52) of studies. The items with the lowest reporting quality were Item 4.2 (setting and context of treatment) at 25.00% (13/52) and Item 5.1 (practitioner background), which was only reported in 3.85% (2/52) of the included studies.

### 3.3 Meta-analysis results

#### 3.3.1 VAS score

**Figure 5:**
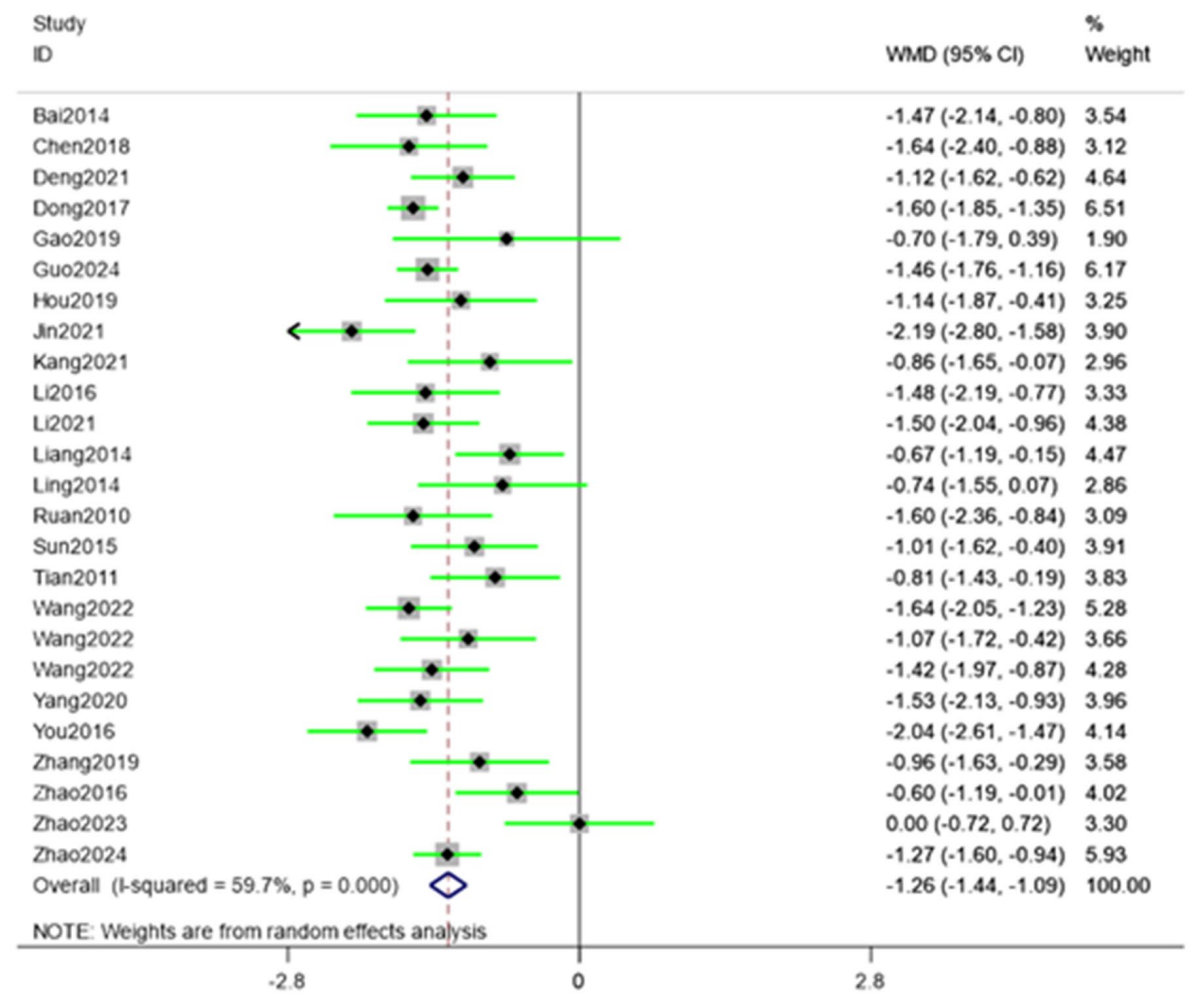
Forest plot showing pooled VAS results comparing acupuncture and control groups.

**Figure 6:**
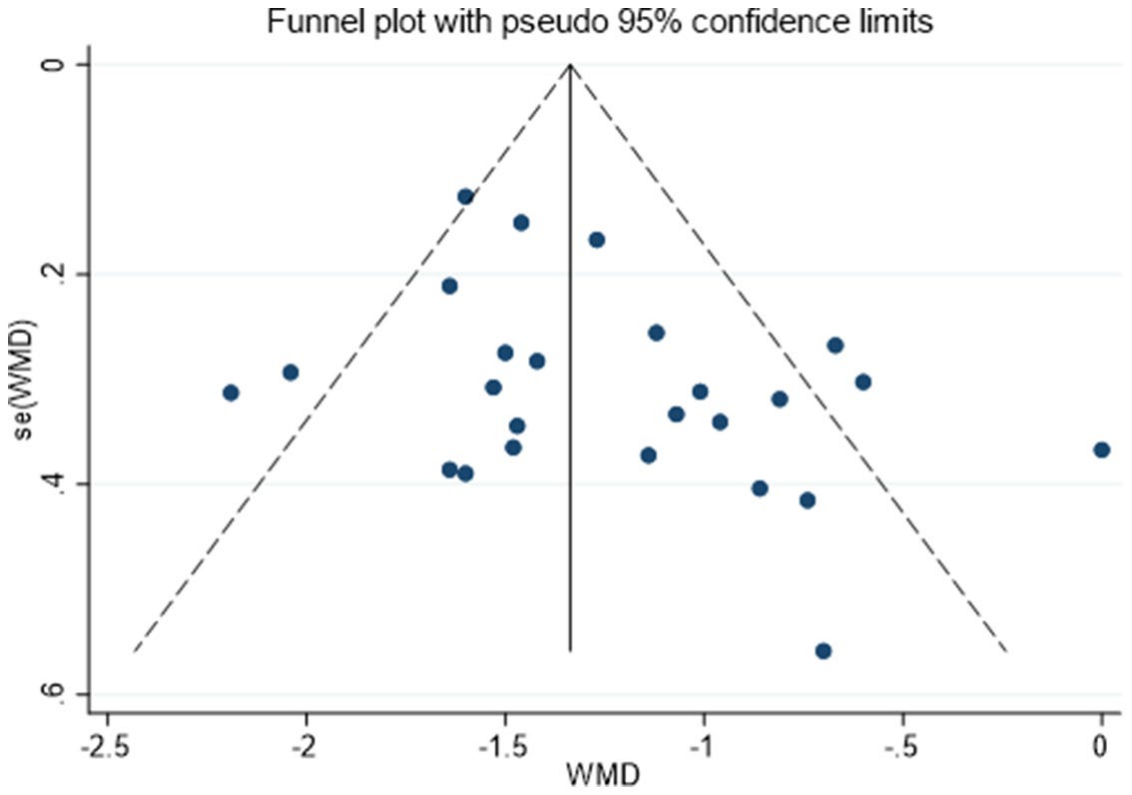
Funnel plot assessing publication bias for VAS outcomes.

Twenty-five randomized trials (30–54) reported VAS outcomes. A random effects model was used, with the mean difference (MD) as the pooled effect size. The random effects model analysis showed that the observation group showed a significant decrease in VAS scores compared to the control group (MD = −1.26, 95% CI [−1.44, −1.09], *I*^2^ = 59.70%). As illustrated in Figure 5, acupuncture produced superior pain relief over the control intervention. The model showed significant amounts of heterogeneity. Sensitivity analysis, publication bias detection, and funnel charts were used to explore heterogeneity. Figure 6 showed no significant publication bias. Begg’s test result *p* = 0.141 and Egger’s test result *p* = 0.054 suggested no significant publication bias (*p* > 0.05). Sensitivity analyses confirmed the robustness of the pooled estimate, as the exclusion of individual studies did not materially alter the overall effect size.

#### 3.3.2 Time of morning stiffness

**Figure 7:**
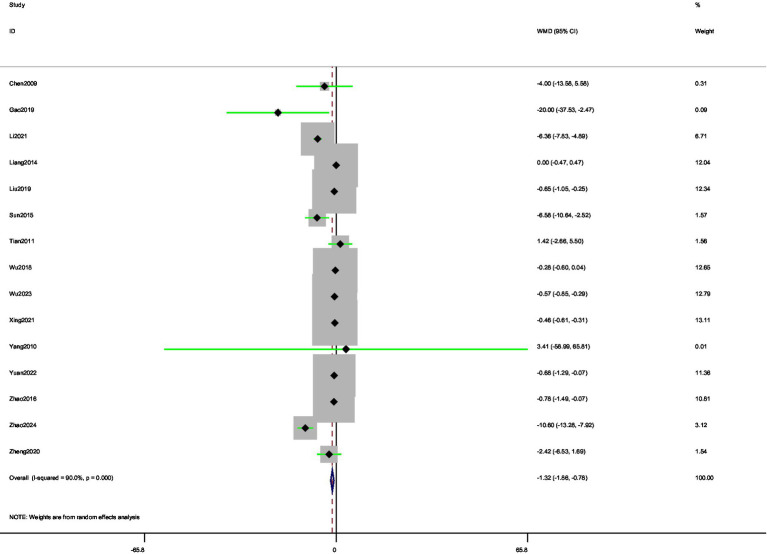
Forest plot of pooled morning stiffness time results comparing acupuncture and control groups.

**Figure 8:**
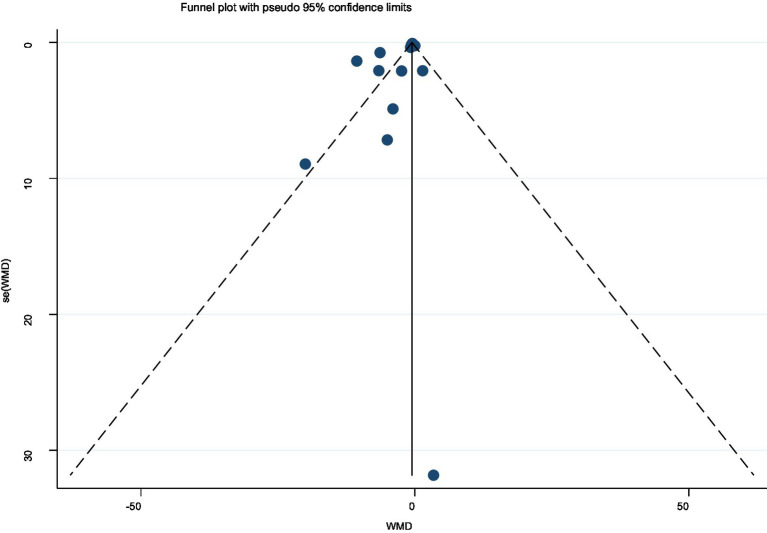
Funnel plot for assessment of publication bias in morning stiffness analysis.

A total of 15 (34, 40, 41, 44, 45, 52–61) studies were involved in reporting morning stiffness time. The random-effects model analysis showed a significant reduction in morning stiffness time compared with control (MD = −1.32, 95% CI [−1.87, −0.78]) (Figure 7). Thus, acupuncture interventions provided superior relief of morning stiffness relative to standard care. Substantial heterogeneity was observed among studies (*I*^2^ = 90.00%). The funnel plot (Figure 8) indicated asymmetry, suggesting possible publication bias for morning stiffness outcomes. Begg’s test did not show significant publication bias (*p* = 0.166), whereas Egger’s test (*p* = 0.037) suggested significant publication bias. Begger’s test did not account for potential missing outcome effects, indicating the presence of publication bias. We used the trim-and-fill method for verification, and the results showed that potential studies were not supplemented, indicating that publication bias was not significant.

#### 3.3.3 Rheumatological indicators (CRP, ESR)

For the pooling of effect sizes, we chose a random-effects model and used mean difference (MD) for the statistics. A total of 43 (30, 31, 34–37, 39, 41–50, 52–77) studies were involved in reporting CRP. Random-effects meta-analysis demonstrated that the observation group exhibited significantly lower CRP levels than the control group (MD = −3.49, 95% CI [−4.12, −2.85]). Substantial between-study heterogeneity was observed (*I*^2^ = 78.90%). The results of Begg’s test (*p* = 0.738) and Egger’s test (*p* = 0.582) were greater than the significance threshold of 0.05, which suggests that the overall study data did not have any significant publication bias problem and that the study results were robust.

A total of 41 (30–32, 34–37, 39, 41–47, 49–55, 57, 59, 61–63, 65, 67–79) studies were involved in reporting ESR. The result suggests that the observation group were highly efficient in reducing the level of ESR, an immune indicator, compared to the control group (MD = −5.36, 95% CI [−6.82, −3.89]). The heterogeneity test results showed that the included studies show substantial heterogeneity (*I*^2^ = 80.7%). The results of Begg’s test at *p* = 0.866 and Egger’s test at *p* = 0.948 suggested no significant publication bias in the overall data of the included studies. The sensitivity analysis results suggested that the study results are robust.

#### 3.3.4 Overall efficacy rate

A total of 37 (32–38, 40, 43, 45–49, 51–55, 57, 58, 60–64, 66–68, 70–73, 75, 76, 78, 79) studies were involved in reporting the overall efficacy rates. Analyses using a random-effects model showed that the efficacy of the observation group was superior to that of the control group (RR = 1.24, 95% CI [1.20, 1.29]). The intervention showed significant advancement in the treatment of AS compared to the control group, as evidenced by the combined results of the total efficacy. The statistically significant heterogeneity between the included studies is not shown in the heterogeneity assessment results (*I*^2^ = 0.00%). The sensitivity analysis results suggested that the study results are robust.

#### 3.3.5 Immunological factors (IL-1, IL-6, IL-17, TNF-α, IgA)

Two studies (33, 80) were involved in reporting IL-1, eight studies (33, 39, 40, 64, 67, 73, 80, 81) were involved in reporting IL-6, four studies (38, 40, 48, 58) were involved in reporting IL-17, 14 studies (39, 42, 47, 48, 56, 58, 64, 65, 67, 73, 74, 78, 80, 81) involvement reported TNF-α, 3 (54, 57, 59). Study involvement reported IgA. Factors IL-1, IL-6, IL-17, TNF-α, and IgA were studied in meta-analyses using a random effects model with SMD as the effect value. The observation group showed a superior decrease in the level of inflammatory factors (IL-1, IL-6, TNF-α, IgA) as compared to the control group. IL-1 (SMD = −2.18, 95% CI [−5.35, −0.10]), *I*^2^ = 97.9%, IL-6 (SMD = −1.28, 95% CI [−2.01, −0.54]), *I*^2^ = 95.00%, IL-17 (SMD = 0.19, 95% CI [−1.77, 2.16]), *I*^2^ = 98.50%, TNF-α (SMD = −1.43, 95% CI [−1.91, −0.96]), *I*^2^ = 93.70%, and IgA (SMD = −1.33, 95% CI [−2.91, 0.25]), *I*^2^ = 95.40%. In the included studies, no significant publication bias in the overall data. Our results showed the sensitivity analyses were stable.

#### 3.3.6 Functional scores (BASDAI, BASFI)

A random effects model is used as the random method, and MD is used as the effect size. A total of 24 (31, 32, 34, 35, 37, 43, 44, 46, 47, 50, 52, 53, 56, 65–68, 70, 72, 76–78, 80, 81) studies were involved in reporting BASDAI. (MD = −1.07, 95% CI [−1.29, −0.84]). This indicates that acupuncture intervention is specific to improving BASDAI. We observed a high degree of heterogeneity between the studies (*I*^2^ = 77.30%) in the heterogeneity test. The low risk of publication bias in the included studies was further confirmed by the results of Begg’s test (*p* = 0.130) and Egger’s test (*p* = 0. 710).

Given that all included studies evaluated BASFI using a unified measurement unit, this study employed mean difference (MD) for meta-analysis to ensure direct clinical interpretability of the results. A total of 23 (31, 32, 34, 35, 37, 43, 44, 46, 47, 50, 52, 54, 56, 58, 65, 66, 68, 72, 76–78, 80, 81) studies were involved in reporting BASFI. BASFI scores of the observer group were significantly lower than those of the control group (MD = −1.06, 95% CI [−1.30, −0.82]) as determined by analyses conducted using a random-effects model. This indicates that acupuncture intervention has a significant effect on improving the effectiveness of BASFI. The heterogeneity test results show a fairly significant degree of heterogeneity between the studies (*I*^2^ = 85.60%). Begg’s test *p* = 0.472 and Egger’s test *p* = 0.448 suggest there is no significant publication bias.

### 3.4 Subgroup analyses

It is divided by intervention, control group type, treatment period, and disease duration with indicators with high heterogeneity (*I*^2^ > 50%) and ≥10 studies (as shown in Table 2).

**Table 2:** Subgroup analyses of outcome indicators by intervention type, control group, treatment duration, and disease course.

Outcome indicators	Subgroup category	Subgroup name	Number of studies	Merge effect value (95% CI)	*p*-value	*I*^2^ (%)
VAS	Intervention subgroup	Acupuncture	8	(−1.52, −1.19)	*p* = 0.000	30.8%
		Acupuncture combined with one type of drugs	6	(−1.06, −1.40)	*p* = 0.000	28.8%
		Acupuncture combined with two types of drugs	16	(−1.40, −0.90)	*p* = 0.000	61.7%
	Control subgroup	Sulfasalazine	8	(−1.24, −0.80)	*P* = 0.000	8.6%
		Sulfasalazine combined with other therapies	11	(−1.50, −1.07)	*P* = 0.000	38.2%
	Treatment subgroup	4w	8	(−1.24, −0.59)	*P* = 0.000	59.0%
		8w	7	(−1.53, −1.04)	*P* = 0.000	39.6%
		12w	6	(−1.63, −1.02)	*P* = 0.000	0.00%
	Disease course subgroup	3–7y	10	(−1.30, −0.76)	*P* = 0.000	51.8%
		>7y	7	(−1.66, −0.88)	*P* = 0.000	65.4%
CRP	Intervention subgroup	Acupuncture	15	(−4.19, −1.22)	*P* = 0.000	80.8%
		Acupuncture combined with one type of drugs	9	(−4.15, −2.29)	*P* = 0.000	77.8%
		Acupuncture combined with two types of drugs	11	(−6.36, −2.29)	*P* = 0.000	75.2%
	Control subgroup	Sulfasalazine	12	(−4.50, −2.74)	*P* = 0.000	41.9%
		Sulfasalazine combined with other therapies	21	(−4.32, −1.66)	*P* = 0.000	78.4%
		Celecoxib combined with other therapies	8	(−5.29, −1.47)	*p* = 0.001	55.3%
		Meloxicam combined with other therapies	6	(−6.53, −2.28)	*P* = 0.000	80.0%
	Treatment subgroup	4w	10	(−5.29, −1.18)	*P* = 0.000	74.3%
		8w	8	(−4.90, −2.75)	*P* = 0.000	58.9%
		12w	15	(−4.48, −2.58)	*P* = 0.000	72.6%
	Disease course subgroup	<3y	7	(−5.41, −0.00)	*p* = 0.050	79.3%
		3–7y	19	(−4.69, −2.85)	*P* = 0.000	77.1%
		>7y	7	(−6.41, −2.31)	*P* = 0.000	80.6%
ESR	Intervention subgroup	Acupuncture	15	(−6.94, −1.05)	*p* = 0.008	87.7%
		Acupuncture combined with one types of drugs	10	(−9.93, −4.00)	*P* = 0.000	81.0%
		Acupuncture combined with two types of drugs	9	(−11.10, −4.26)	*P* = 0.000	81.7%
	Control subgroup	Sulfasalazine	14	(−5.40, −3.15)	*P* = 0.000	24.0%
		Sulfasalazine combined with other therapies	19	(−8.24, −3.28)	*P* = 0.000	84.3%
		Celecoxib combined with other therapies	7	(−13.27, −2.29)	*p* = 0.006	84.5%
		Meloxicam combined with other therapies	6	(−8.30, −3.96)	*P* = 0.000	40.3%
	Treatment subgroup	4w	10	(−9.16, −0.92)	*p* = 0.016	89.4%
		8w	9	(−6.16, −3.33)	*P* = 0.000	20.0%
		12w	13	(−8.75, −3.62)	*P* = 0.000	82.5%
	Disease course subgroup	<3y	9	(−5.51, −3.14)	*P* = 0.000	0.0%
		3–7y	21	(−8.19, −4.15)	*P* = 0.000	81.1%
		>7y	6	(−14.24, −4.99)	*P* = 0.000	81.9%
BASDAI	Intervention subgroup	Acupuncture	6	(−1.06, −0.32)	*P* = 0.001	68.7%
		Acupuncture combined with one type of drugs	6	(−1.29, −0.82)	*P* = 0.000	0.0%
		Acupuncture combined with two types of drugs	7	(−1.46, −0.85)	*P* = 0.000	44.8%
	Control subgroup	Sulfasalazine	6	(−1.29, −0.82)	*P* = 0.000	0.0%
		Sulfasalazine combined with other therapies	12	(−1.30, −0.74)	*P* = 0.000	66.0%
		Celecoxib combined with other therapies	7	(−1.78, −0.66)	*P* = 0.000	87.3%
	Treatment subgroup	4w	8	(−1.21, −0.65)	*P* = 0.000	20.5%
		8w	6	(−1.48, −0.74)	*P* = 0.000	70.1%
		12w	5	(−1.59, −0.78)	*P* = 0.000	71.8%
	Disease course subgroup	3–7y	10	(−1.32, −0.86)	P = 0.000	52.7%
		>7y	6	(−1.88, −0.70)	*P* = 0.000	86.7%
BASFI	Intervention subgroup	Acupuncture	6	(−1.83, −0.72)	*P* = 0.000	95.6%
		Acupuncture combined with one type of drugs	6	(−1.04, −0.59)	*P* = 0.000	0.0%
		Acupuncture combined with two types of drugs	8	(−1.33, −0.66)	*P* = 0.000	54.4%
	Control subgroup	Sulfasalazine	6	(−1.04, −0.59)	*P* = 0.000	0.0%
		Sulfasalazine combined with other therapies	12	(−1.40, −0.88)	*P* = 0.000	65.1%
		Celecoxib combined with other therapies	5	(−1.60, −0.47)	*P* = 0.000	95.6%
	Treatment subgroup	4w	6	(−1.60, −0.58)	*P* = 0.000	76.5%
		8w	6	(−1.61, −0.87)	*P* = 0.000	56.9%
		12w	5	(−1.59, −0.78)	*p* = 0.0066	71.9%
	Disease course subgroup	<3y	5	(−1.15, −0.64)	*P* = 0.000	0.0%
		3–7y	13	(−1.34, −0.71)	*P* = 0.000	91.5%
		>7y	4	(−1.53, −0.50)	*P* = 0.000	61.9%

For the pain outcome, as measured by the Visual Analog Scale (VAS), treatment duration was the most significant factor influencing heterogeneity. The analysis showed that heterogeneity was completely resolved in studies with a 12-week treatment duration (*I*^2^ = 0.0%, *p* = 0.436), which demonstrated a significant MD of −1.33 (95% CI [−1.63, −1.02]). Low, non-significant heterogeneity was also observed in the 8-week treatment subgroup (*I*^2^ = 39.6%, *p* = 0.127). In contrast, substantial heterogeneity was observed in the 4-week treatment subgroup (*I*^2^ = 59.0%) and when acupuncture was combined with two types of drugs (*I*^2^ = 61.7%).

The analysis of inflammatory markers presented a more complex picture. For ESR, heterogeneity was resolved in patients with a disease course of less than 3 years (*I*^2^ = 0.0%) and was substantially reduced during an 8-week treatment course (*I*^2^ = 20.0%). In contrast, heterogeneity for CRP remained moderate to high across all subgroup analyses (*I*^2^ > 41%), indicating that its variability is multifactorial and not easily explained by a single factor. The most consistent results for CRP were found in studies using sulfasalazine monotherapy as a control, though heterogeneity was still moderate (*I*^2^ = 41.9%).

For the functional outcomes BASDAI and BASFI, heterogeneity was primarily driven by the study design. The variability was completely resolved (*I*^2^ = 0.0%) in subgroups where acupuncture was combined with a single conventional drug. For BASFI specifically, heterogeneity was also eliminated in studies including patients with an early disease course of less than 3 years (*I*^2^ = 0.0%). Conversely, the highest levels of heterogeneity for these functional outcomes were consistently observed in the acupuncture monotherapy subgroups (BASDAI: *I*^2^ = 68.7%; BASFI: *I*^2^ = 95.6%). This strongly suggests that the initial high heterogeneity was a direct result of inconsistent intervention and control protocols across studies.

### 3.5 GRADE approach results

We performed a GRADE approach on the results of this meta-analysis and found that the outcomes of all included studies were of low to moderate quality, which to some extent reflects the limitations of the current research. For details, please refer to Table 3 Grade approach results.

**Table 3:** Grade approach results.

Certainty assessment							
No of studies	Study type	Risk of bias	Inconsistency	Indirectness	Imprecision	Other considerations	Certainty
VAS score							
25	Randomized clinical trials	Not serious	Serious	Not serious	Not serious	None	Moderate ⨁⨁⨁◯
Time of morning stiffness							
15	Randomized clinical trials	Not serious	Serious	Not serious	Serious	Serious	Very low ⨁◯◯◯
Overall efficacy rate							
37	Randomized clinical trials	Not serious	Not serious	Not serious	Not serious	Serious	Moderate ⨁⨁⨁◯
CRP							
43	Randomized clinical trials	Not serious	Serious	Not serious	Not serious	None	Moderate ⨁⨁⨁◯
ESR							
41	Randomized clinical trials	Not serious	Serious	Not serious	Not serious	None	Moderate ⨁⨁⨁◯
BASDAI							
24	Randomized clinical trials	Not serious	Serious	Not serious	Not serious	None	Moderate ⨁⨁⨁◯
BASFI							
23	Randomized clinical trials	Not serious	Serious	Not serious	Not serious	None	Moderate ⨁⨁⨁◯
IL-1							
2	Randomized clinical trials	Not serious	Serious	Not serious	Serious	Serious	Very low ⨁◯◯◯
IL-6							
8	Randomized clinical trials	Not serious	Serious	Not serious	Serious	Serious	Very low ⨁◯◯◯
IL-17							
4	Randomized clinical trials	Not serious	Serious	Not serious	Serious	Serious	Very low ⨁◯◯◯
TNF-α							
14	Randomized clinical trials	Not serious	Serious	Not serious	Not serious	Serious	Low ⨁⨁◯◯
IgA							
3	Randomized clinical trials	Not serious	Serious	Not serious	Serious	Serious	Very low ⨁◯◯◯

### 3.6 Sensitivity analyses

Sensitivity analyses confined to the 20 trials rated as low risk of bias (RoB 2) preserved both the direction and magnitude of the pooled estimates for all outcomes except interleukin-6. Effect sizes for VAS score, morning stiffness duration, CRP, ESR, overall efficacy rate, TNF-α, BASDAI and BASFI remained statistically congruent with the primary analysis. The summary estimate for IL-6, however, was no longer statistically significant, a shift ascribed to the limited number of low-bias trials available for this comparison (*n* = 2), which eroded statistical power and expanded the confidence interval. Insufficient numbers of low-bias studies precluded meta-analysis of IL-1, IL-17 and IgA (each *n* < 2).

## 4 Discussion

### 4.1 Main findings and summary

The efficacy of acupuncture in the treatment of AS is comprehensively assessed in 63 studies that are included in this systematic review and meta-analysis. The results demonstrated that acupuncture therapy shows significant advantages over the control group in the areas of reducing inflammation, reducing pain, and improving function. It offers new ideas and evidence for the clinical treatment of AS. However, the risk of bias and heterogeneity issues in the studies deserve in-depth discussion. According to the Cochrane tool, most studies were judged at moderate risk of bias, mainly concerning allocation concealment and blinding. These issues may introduce selection, performance, and detection biases, thereby undermining the credibility of the results. Acupuncture demonstrated significant benefits in patients with AS, evidenced by reductions in inflammatory markers (CRP, ESR) and improvements in pain (VAS), disease activity (BASDAI), and physical function (BASFI). The decline in CRP and ESR suggests an anti-inflammatory effect that may underpin the therapy’s ability to modify disease progression. Concurrently, the alleviation of pain and enhancement of functional capacity, reflected by the VAS, BASDAI, and BASFI scores, highlight its promise in managing core symptoms and improving patients’ quality of life.

A key methodological issue in this review is that we pooled studies despite substantial clinical heterogeneity in acupuncture parameters, including acupoint prescriptions, needling technique (manual vs. electroacupuncture), stimulation intensity, needle retention time, session frequency and total treatment duration. This decision was driven by both pragmatic and precedent-based considerations. First, the field of acupuncture research has traditionally used meta-analytic pooling of heterogeneous protocols to estimate an average effect of acupuncture as a treatment modality, as exemplified by large IPD meta-analyses of chronic pain and numerous condition-specific systematic reviews (82–85). In these influential works, diverse acupoint combinations and dosing regimens were synthesized under a random-effects framework, with the understanding that the pooled effect reflects a class effect of acupuncture rather than a single standardized protocol (82, 84, 86). Second, the reporting of acupuncture interventions remains suboptimal in many trials, despite the availability of the STRICTA (STandards for Reporting Interventions in Clinical Trials of Acupuncture) guidelines (87, 88).

### 4.2 Heterogeneity and publication bias

These findings highlight the importance of accounting for publication bias, especially for outcomes with large effect sizes. Future research should aim to include unpublished data to further reduce bias.

Moderate to high heterogeneity (*I*^2^) was observed for most outcomes, primarily attributable to clinical and methodological diversity rather than chance. Subgroup analyses substantially reduced heterogeneity for BASDAI, BASFI, and ESR when isolating specific intervention designs (e.g., acupuncture combined with a single drug), treatment durations (12 weeks for VAS, 8 weeks for ESR), or early-stage disease populations (<3 years). In contrast, heterogeneity for CRP persisted across all subgroups. The observed heterogeneity complicates the assessment of publication bias, as asymmetric effect distributions may arise from clinical/methodological variation rather than selective publication. Trim-and-fill analysis indicated no significant publication bias, supporting that methodological diversity, not missing studies, poses the primary threat to validity. Future studies should standardize protocols and control groups to enhance evidence consistency.

A central challenge when interpreting our pooled results lies in the heterogeneity of baseline disease severity/activity among included trials. According to recent European recommendations for axial spondyloarthritis (axSpA, which includes AS) by Assessment of SpondyloArthritis international Society (ASAS) and European Alliance of Associations for Rheumatology (EULAR), optimal management decisions (e.g., whether to escalate to biologic therapy) depend on a composite assessment of disease activity — combining patient-reported symptoms, inflammatory biomarkers (e.g., CRP), and imaging (MRI or radiographic sacroiliitis) (89). The guideline explicitly recommends that biologic or targeted synthetic DMARDs be considered in patients with high disease activity (e.g., elevated CRP, MRI evidence of active inflammation or radiographic sacroiliitis) and inadequate response to at least two NSAIDs.

Without stratifying by baseline disease severity, pooled estimates may obscure differential effects. For example, acupuncture might be more effective (or detectable) in patients with lower-to-moderate disease activity (where pain and stiffness dominate) than in those with high inflammatory burden, structural damage, or advanced radiographic AS. Alternatively, immunomodulatory effects (reflected in biomarkers) might differ depending on whether patients are in an early inflammatory phase (with active sacroiliitis) or in a later structural/damage-predominant phase. Therefore, our pooled effect should be interpreted as an average across a clinically heterogeneous AS population, rather than representing a single uniform “standardized” effect applicable to all AS patients.

### 4.3 Explanation of the mechanism of acupuncture

Multiple studies have demonstrated that acupuncture stimulation of specific acupoints can modulate immune function (90). The stimulation signals are transmitted via the vagus nerve to the central nervous system, which in turn activates the neuro-immune-endocrine axis, thereby regulating immune cell activity. Specifically, acupuncture has been shown to enhance regulatory T-cell (Treg) expansion and inhibit Th17 differentiation in lymphoid tissues (91, 92), leading to decreased production of pro-inflammatory cytokines (e.g., IL-6, TNF-α) and increased secretion of anti-inflammatory mediators (e.g., IL-10). It can reduce the level of pro-inflammatory factors, such as IL-6 and TNF-α, and increase the production of anti-inflammatory factors (93, 94), thus exerting analgesic and anti-inflammatory effects. Experimental animal studies confirm that needling at defined acupoints significantly lowers serum IL-6 and TNF-α concentrations (95). Mechanistically, these effects are attributed to the activation of endogenous anti-inflammatory signaling cascades. The hypothalamic–pituitary–adrenal axis (HPA axis) is a complex neuroendocrine system (96). The HPA axis synthesizes and secretes cortisol, which forms a complex with the glucocorticoid receptor (GR) (97) and inhibits the production of a variety of pro-inflammatory mediators while all at once producing the production of anti-inflammatory mediators. Acupuncture stimulates hypothalamic CRH secretion, thereby activating the HPA axis. This response restores cytokine homeostasis by reducing excessive inflammation and enhancing anti-inflammatory signaling, which correlates with the alleviation of joint pain and functional impairment (98, 99).

From a TCM perspective, acupoint selection in the included studies can be understood within the framework of syndrome differentiation (bianzheng), including meridian differentiation, zang–fu differentiation and the eight-principle patterns. Treatment based on meridian differentiation emphasizes identifying the most affected meridian(s), then choosing points along the corresponding channels to restore the flow of qi and blood (100–102). Based on the basic principles of traditional Chinese medicine, such as “tonifying the kidney and strengthening the Du meridian, dispelling cold and dampness, promoting blood circulation and unblocking collaterals,” and the theory of “meridians passing through, indications reaching,” the treatment of ankylosing spondylitis mainly selects acupoints such as Jiaji acupoint, Shenshu acupoint, Mingmen acupoint, and the Foot Sun Bladder Meridian.

Importantly, the tendency toward flexible, individualized acupoint prescriptions in routine practice was partly reflected in the trials we included, in which acupuncturists often adjusted one or two points based on symptom severity or concurrent TCM patterns. This is consistent with data-mining and network analyses of acupuncture prescriptions, which show that acupoints cluster into reproducible combinations and meridian modules rather than being used in isolation (100, 103).

### 4.4 Comparison with previous studies or guidelines

Prior investigations indicate that integrating acupuncture with conventional pharmacotherapy for AS can alleviate pain, lower inflammatory biomarkers, and ameliorate clinical symptoms. The latest Cochrane review (104) evidence quality is moderate to high, the intervention program is limited to medication, there is a single treatment option, and the recommendation for acupuncture combined with Western medicine is low. There is a degree of recognition of acupuncture combined with Western medicine treatment in Chinese guideline recommendations (105), but the exact strength of the recommendation may vary depending on the level of evidence. These guidelines are further informed by traditional Chinese medicine practices and regional clinical data. In contrast, European and North American guidelines are primarily driven by the indication spectra of pharmacotherapies. Although these pharmacological recommendations cover a broader range of options, they are often supported by limited evidence and expert consensus rather than high-level trial data. Consequently, acupuncture rarely features prominently in these guidelines, and when cited, its recommendation is relatively weak. In sharp contrast to Cao’s perspective (106), we shift the focus to the effect of acupuncture on immune markers, specifically highlighting that it exerts its efficacy by modulating inflammatory factors, thereby ameliorating the symptoms of AS.

While established Minimal Clinically Important Difference (MCID) values for these outcomes are not explicitly reported in the literature, the clinical relevance of the improvements observed in this meta-analysis can be contextualized by comparing them with effect sizes from other recognized interventions. For example, a prior study (107) on combination anti-rheumatic therapy reported a VAS improvement with a mean difference (MD) of −0.74 in patients with AS. The greater reduction observed in our analysis (MD = −1.26) suggests that acupuncture may offer a comparable, if not superior, magnitude of pain relief. Similarly, for morning stiffness duration, another reference study (108) showed a modest reduction (MD = −0.28), whereas the effect size in our meta-analysis was substantially larger (MD = −1.32). These comparisons support the clinical relevance of acupuncture in improving both pain and morning stiffness in AS, even in the absence of formally defined MCID thresholds.

Despite the established role of VAS and morning stiffness in monitoring AS disease activity and treatment response, no specific MCID values for these endpoints have been reported in studies published over the past 5 years, even though significant changes in these parameters are consistently observed following effective treatment.

### 4.5 Limitations and future research implications

This study has several limitations that warrant caution when interpreting the findings. The majority of included trials were conducted in China, reflecting considerable regional research interest but also introducing potential biases related to local practice patterns and culture, which may limit the generalizability of results to other populations. Methodologically, the included studies did not include the training background, certification level or years of clinical experience of relevant acupuncture and moxibustion. The lack of these details limited the repeatability of the intervention and the universality of the results. ROB shows some moderate risk bias, so the research design needs to be rigorous. The certainty of evidence, as per the GRADE framework, was low to moderate for primary outcomes, largely due to substantial heterogeneity, risk of bias, and imprecision in effect estimates. Sensitivity analyses confirmed that these methodological weaknesses consistently undermined the robustness of the conclusions. Incomplete documentation of acupoint selection rationale, needling depth, stimulation technique, and co-interventions limits the feasibility of fine-grained subgroup or component analyses in meta-analysis. In our review, these reporting limitations constrained our ability to pre-specify and reliably evaluate more detailed categories of acupuncture “dose” or style, even when clinical heterogeneity was apparent.

We adopted a random-effects pooling strategy, complemented—where data allowed—by exploratory subgroup analyses and sensitivity analyses excluding outlier trials. This approach is aligned with methodological discussions in the acupuncture literature, which acknowledge that, under current evidence structures, pooling heterogeneous acupuncture protocols is often unavoidable but should be interpreted cautiously. We recommend acupuncture as a complementary therapy for pain and functional limitation in AS. Future research should prioritize rigorously designed, multicenter RCTs with larger sample sizes, standardized protocols, and longer follow-up to better establish long-term efficacy and safety.

## 5 Conclusion

Acupuncture shows promise as an adjunctive therapy for AS, with this analysis suggesting potential benefits for clinical symptoms and inflammatory biomarkers. The findings indicate that combining basic medication with acupuncture-related therapies was associated with an alleviation of AS symptoms, including joint pain and stiffness, as well as reductions in the levels of pro-inflammatory cytokines such as IL-6 and TNF-*α*. However, these findings must be interpreted with considerable caution. The substantial statistical heterogeneity observed across most outcomes and the potential for publication bias in some analyses limit the generalizability of these results and highlight the need for more methodologically rigorous and uniform research to establish a reliable, evidence-based foundation for acupuncture in the treatment of AS.

## Data Availability

The original contributions presented in the study are included in the article/Supplementary material, further inquiries can be directed to the corresponding authors.

## References

[1] Raychaudhuri SP Deodhar A. The classification and diagnostic criteria of ankylosing spondylitis. J Autoimmun. (2014) 48:128–33. doi: 10.1016/j.jaut.2014.01.015, 24534717

[2] Sieper J Poddubnyy D. Axial spondyloarthritis. Lancet. (2017) 390:73–84. doi: 10.1016/s0140-6736(16)31591-4, 28110981

[3] Dean LE Jones GC MacDonald AG Downham C Sturrock RD Macfarlane GJ. Global prevalence of ankylosing spondylitis. Rheumatology. (2014) 53:650–7. doi: 10.1093/rheumatology/ket387, 24324212

[4] Wright GC Kaine J Deodhar A. Understanding differences between men and women with axial spondyloarthritis. Semin Arthritis Rheum. (2020) 50:687–94. doi: 10.1016/j.semarthrit.2020.05.005, 32521322

[5] Ward MM Deodhar A Gensler LS Dubreuil M Yu D Khan MA . 2019 update of the American College of Rheumatology/spondylitis Association of America/Spondyloarthritis research and treatment network recommendations for the treatment of ankylosing spondylitis and nonradiographic axial spondyloarthritis. Arthritis Rheumatol. (2019) 71:1599–613. doi: 10.1002/art.41042, 31436036 PMC6764882

[6] McVeigh CM Cairns AP. Diagnosis and management of ankylosing spondylitis. BMJ. (2006) 333:581–5. doi: 10.1136/bmj.38954.689583.DE, 16974012 PMC1570004

[7] van der Heijde D Landewé R. Imaging in spondylitis. Curr Opin Rheumatol. (2005) 17:413–7. doi: 10.1097/01.bor.0000163195.48723.2d, 15956837

[8] Boonen A Sieper J Van Der Heijde D Dougados M Bukowski JF Valluri S . The burden of non-radiographic axial spondyloarthritis. Semin Arthritis Rheum. (2015) 44:556–62. doi: 10.1016/j.semarthrit.2014.10.009, 25532945

[9] Tam L-S Gu J Yu D. Pathogenesis of ankylosing spondylitis. Nat Rev Rheumatol. (2010) 6:399–405. doi: 10.1038/nrrheum.2010.79 20517295

[10] Bleil J Maier R Hempfing A Schlichting U Appel H Sieper J . Histomorphologic and histomorphometric characteristics of zygapophyseal joint remodeling in ankylosing spondylitis. Arthritis Rheumatol. (2014) 66:1745–54. doi: 10.1002/art.38404, 24574301

[11] Dougados M Dijkmans B Khan M Maksymowych W Van Der Linden S Brandt J. Conventional treatments for ankylosing spondylitis. Ann Rheum Dis. (2002) 61:iii40–50. doi: 10.1136/ard.61.suppl_3.iii40, 12381510 PMC1766726

[12] Dinarello CA. Anti-inflammatory agents: present and future. Cell. (2010) 140:935–50. doi: 10.1016/j.cell.2010.02.043, 20303881 PMC3752337

[13] Kelly RB Willis J. Acupuncture for pain. Am Fam Physician. (2019) 100:89–96. 31305037

[14] Wang M Liu W Ge J Liu S. The immunomodulatory mechanisms for acupuncture practice. Front Immunol. (2023) 14:1147718. doi: 10.3389/fimmu.2023.1147718, 37090714 PMC10117649

[15] Fan AY Miller DW Bolash B Bauer M McDonald J Faggert S . Acupuncture’s role in solving the opioid epidemic: evidence, cost-effectiveness, and care availability for acupuncture as a primary, non-pharmacologic method for pain relief and management–White paper 2017. J Integr Med. (2017) 15:411–25. doi: 10.1016/s2095-4964(17)60378-9 29103410

[16] Chen T Zhang WW Chu Y-X Wang Y-Q. Acupuncture for pain management: molecular mechanisms of action. Am J Chin Med. (2020) 48:793–811. doi: 10.1142/s0192415x20500408, 32420752

[17] Chan MWC Wu XY Wu JCY Wong SYS Chung VCH. Safety of acupuncture: overview of systematic reviews. Sci Rep. (2017) 7:3369. doi: 10.1038/s41598-017-03272-0, 28611366 PMC5469776

[18] Bae R Kim HK Lu B Ma J Xing J Kim HY. Role of hypothalamus in acupuncture’s effects. Brain Sci. (2025) 15:72. doi: 10.3390/brainsci15010072, 39851439 PMC11763592

[19] Li N Guo Y Gong Y Zhang Y Fan W Yao K . The anti-inflammatory actions and mechanisms of acupuncture from acupoint to target organs via neuro-immune regulation. J Inflamm Res. (2021) 14:7191–224. doi: 10.2147/jir.S341581, 34992414 PMC8710088

[20] Ye MX Li JM Zhang XJ Zheng BL Chen JL Zhang MH. Clinical observation on the wrist-ankle acupuncture combined with intradermal needling in the treatment of ankylosing spondylitis. J Guangzhou Univ Tradit Chin Med. (2022) 39:1562–7. doi: 10.13359/j.cnki.gzxbtcm.2022.07.018

[21] Hou HK Xiong DC Li JM. Efficacy of Wenyang Tongdu acupuncture combined with internal heated needling in treating AS of cold-dampness obstruction and its influence to serum levels of ESR, CRP and RF. J Clin Acupunct Moxibustion. (2021) 37:18–21. doi: 10.19917/j.cnki.1005-0779.021217

[22] Wang ZM Li WQ Tian XM Chen WY Wang JJ. Observation on clinical effects of needle-knife combined with etanercept in improving spinal dysfunction of ankylosing spondylitis. West J Tradit Chin Med. (2016) 29:119–22. doi: 10.3969/j.issn.1004-6852.2016.01.037

[23] Xuan Y Huang H Huang Y Liu D Hu X Geng L. The efficacy and safety of simple-needling therapy for treating ankylosing spondylitis: a systematic review and meta-analysis of randomized controlled trials. Evid Based Complement Alternat Med. (2020) 2020:4276380. doi: 10.1155/2020/4276380, 32617106 PMC7306850

[24] Page MJ McKenzie JE Bossuyt PM Boutron I Hoffmann TC Mulrow CD . The PRISMA 2020 statement: an updated guideline for reporting systematic reviews. BMJ. (2021) 372:n71. doi: 10.1136/bmj.n71, 33782057 PMC8005924

[25] Van Der Linden S Valkenburg HA Cats A. Evaluation of diagnostic criteria for ankylosing spondylitis. Arthritis Rheum. (1984) 27:361–8. doi: 10.1002/art.1780270401, 6231933

[26] Sieper J Van Der Heijde D Landewé R Brandt J Burgos-Vagas R Collantes-Estevez E . New criteria for inflammatory back pain in patients with chronic back pain: a real patient exercise by experts from the assessment of SpondyloArthritis international society (ASAS). Ann Rheum Dis. (2009) 68:784–8. doi: 10.1136/ard.2008.101501, 19147614

[27] Zochling J. Measures of symptoms and disease status in ankylosing spondylitis. Arthritis Care Res. (2011) 63:S47–58. doi: 10.1002/acr.20575, 22588768

[28] Sterne JAC Savović J Page MJ Elbers RG Blencowe NS Boutron I . RoB 2: a revised tool for assessing risk of bias in randomised trials. BMJ. (2019) 366:l4898. doi: 10.1136/bmj.l4898 31462531

[29] Al Duhailib Z Granholm A Alhazzani W Oczkowski S Belley-Cote E Møller MH. GRADE pearls and pitfalls—part 1: systematic reviews and meta-analyses. Acta Anaesthesiol Scand. (2024) 68:584–92. doi: 10.1111/aas.14386, 38351600

[30] Bai Y Gao Y Guo HQ Lou YQ Zhou ZP. Clinical study on needling points mainly along the spinal column to relieve pain and improve activity of patients with ankylosing spondylitis. Arthritis Rheum. (2014) 3:12–3. doi: 10.3969/j.issn.2095-4174.2014.04.003

[31] Chen QH Ge JR Yin Q. CT-guided acupotomy release combined with medication for 30 cases of ankylosing spondylitis. Fujian J Tradit Chin Med. (2018) 49:9–11. doi: 10.13260/j.cnki.jfjtcm.011582

[32] Gui-yi D Jian-hui H Xing-mu Z Jia-wei H Zeng-sheng W Yue-hui Q. Effects of acupuncture plus spinal manipulations on physical functioning and biochemical indicators in patients with ankylosing spondylitis. J Acupunct Tuina Sci. (2021) 19:206–12. doi: 10.1007/s11726-021-1241-0

[33] Dong JL Li YB Cai Y. Observations on the therapeutic effect of electrothermal acupuncture on ankylosing spondylitis and its impact on IL-1 and IL-6. Shanghai J Acupunct Moxibustion. (2017) 36:444–8. doi: 10.13460/j.issn.1005-0957.2017.04.0444

[34] Gao TZ Huang GF. Clinical observation of warm needling moxibustion at Jiaji point combined with western medicine in treatment of ankylosing spondylitis. Hubei J Tradit Chin Med. (2019) 41:17–9.

[35] Guo CL Wu J Zhang W Yan XQ. Therapeutic observation of exercise-based acupuncture combined with medication and functional training for ankylosing spondylitis. Shanghai J Acupunct Moxibustion. (2024) 43:400–4. doi: 10.13460/j.issn.1005-0957.2023.13.0016

[36] Hou YX Li M Yao J Wu JM Liu D. Clinical observation of acupuncture therapy of combination of dredging governor vessel and regulating mentality and acupoint injection in treating ankylsing spondylitis. Shandong J Tradit Chin Med. (2019) 38:770–3. doi: 10.16295/j.cnki.0257-358x.2019.08.014

[37] Jin ZH Zhang QY Gu GZ Zhang L Zhou J. Clinical observation on 25 cases of active ankylosing spondylitis treated by celecoxib combined with Du Meridian moxibustion and pressing needle. Rheum Arthritis. (2021) 10:8–10. doi: 10.3969/j.issn.2095-4174.2021.02.003

[38] Kang ZJ. Clinical observation of comprehensive acupuncture treatment for ankylosing spondylitis. Guangming J Chin Med. (2021) 36:4034–5. doi: 10.3969/j.issn.1003-8914.2021.23.037

[39] Li T Zhang XJ Ye MX Zheng BL Guo QH. Effect of floating-needle plus tube-embedding therapy on tumor necrosis factor-α and interleukin-6 in patients with ankylosing spondylitis. New Chin Med. (2016) 48:155–7. doi: 10.13457/j.cnki.jncm.2016.08.067

[40] Li QR Zhang WZ. Clinical observation of acupuncturing the points of Du channel combined with western medicine in treating ankylosing spondylitis. Chinas Naturop. (2021) 29:107–10. doi: 10.19621/j.cnki.11-3555/r.2021.1340

[41] Liang YY Guo YM Feng H Gu JQ Zhou SL. Clinical observations on combined use of acupuncture and medicine for the treatment of ankylosing spondylitis. Shanghai J Acupunct Moxibustion. (2014) 33:548–51. doi: 10.13460/j.issn.1005-0957.2014.06.0548

[42] Ling X Zhu YY. Effect of catgut implantation at acupoint on the content of tumor necrosis factor α of patients with ankylosing spondylitis. Chin J Integr Tradit West Med. (2014) 34:284–6. doi: 10.7661/CJIM.2014.03.0284 24758077

[43] Ruan YJ Wang J Liu J. Clinical efficacy analysis of CT-guided sacroiliac joint acupotomy release for early and middle-stage ankylosing spondylitis. New Chin Med. (2010) 42:99–101. doi: 10.13457/j.cnki.jncm.2010.12.062

[44] Sun GW Wang L. Clinical comparative observation of fire-needle therapy and routine therapy for ankylosing spondylitis. Chinas Naturop. (2015) 23:30–2. doi: 10.19621/j.cnki.11-3555/r.2015.02.021

[45] Tian YS Wang LS Wang XY Sun WQ. Clinical observation on ankylosing spondylitis treated with acupoint catgut embedding combined vessel pricking therapy. Chin Acupunct Moxibustion. (2011) 31:601–4. doi: 10.13703/j.0255-2930.2011.07.010, 21823280

[46] Wang WY Zhao C Zhang M Zhang JJ Yu HW. Clinical observation on treating ankylosing spondylitis of the kidney deficiency and Du-Meridian cold type by Liji-acupuncture plus Du-moxibustion. Clin J Chin Med. (2022) 14:45–8. doi: 10.3969/j.issn.1674-7860.2022.04.009

[47] Wang ZR Li YD Zhang J. Effect of akupotomye lumbar-abdominal simultaneous regulation combined with sulfasalazine on ankylosing spondylitis. Chin J Pract Med. (2022) 49:120–3. doi: 10.3760/cma.j.cn115689-20220112-00157

[48] Wang DJ Yang B. Efficacy observation of acupuncture combined with auricular point sticking for early-to-moderate-stage ankylosing spondylitis. Shanghai J Acupunct Moxibustion. (2022) 41:562–8. doi: 10.13460/j.issn.1005-0957.2022.06.0562

[49] Yang LH Ouyang BS. Clinical observation of warming acupuncture and moxibustion in treating active stage of ankylosing spondylitis with kidney deficiency and governor vessel cold type. Chinas Naturop. (2020) 28:26–8. doi: 10.19621/j.cnki.11-3555/r.2020.1912

[50] You YQ Chen CX Xu CC Li ZQ Cai MM Liu LQ. Clinical observation on the treatment of ankylosing spondylitis with sacroiliac joint acupotomy. Rheum Arthritis. (2016) 5:14–8. doi: 10.3969/j.issn.2095-4174.2016.03.003

[51] Zhang HH Wang ZY Zhang ZB Li P. Clinical observation on moxibustion at Du Meridian combined with acupuncture in treating ankylosing spondylitis. Chin Med Mod Distance Educ China. (2019) 17:91–3. doi: 10.3969/j.issn.1672-2779.2019.10.035

[52] Zhao F Fan SQ Chen J Ke SH. Observation of the efficacy of warm-needle moxibustion combined with medication in the treatment of enthesitis in ankylosing spondylitis. Shanxi J Tradit Chin Med. (2016) 32:31–2. doi: 10.3969/j.issn.1000-7156.2016.11.015

[53] Zhao MY Lin C. Observation of the efficacy of acupuncture of Zhuang medicine in the treatment of ankylosing spondylitis. J Guangxi Univ Chin Med. (2023) 26:14–7. doi: 10.3969/j.issn.2095-4441.2023.03.004

[54] Zhao Y Zhang Y Dou CZ Chen Y Geng J Sun Y. Clinical study of straight-side acupuncture in treatment of ankylosing spondylitis. J Clin Acupunct Moxibustion. (2024) 40:43–7. doi: 10.19917/j.cnki.1005-0779.024072

[55] Chen YH. Observation of the efficacy of warm-needle moxibustion combined with functional exercise in the treatment of ankylosing spondylitis. J Emerg Tradit Chin Med. (2009) 18:1611–34. doi: 10.3969/j.issn.1004-745X.2009.10.026

[56] Liu BY. Effect of acupuncture combined with conventional drugs on ankylosing spondylitis. China Mod Med. (2019) 26:21–24,33. doi: 10.3969/j.issn.1674-4721.2019.02.007

[57] Wu ZB Zhang Z. Clinical efficacy of 30 cases about filiform-fire needle combined with abdominal acupuncture therapy in ankylosing spondylitis. J Zhejiang Chin Med Univ. (2018) 42:247–50. doi: 10.16466/j.issn1005-5509.2018.03.019

[58] Wu J Liang Y Li M Yu WJ. Efficacy of kinematic acupuncture combined with western medicine in ankylosing spondylitis and its effect on BASFI and BASDAI score. Shanghai J Acupunct Moxibustion. (2023) 42:56–60. doi: 10.13460/j.issn.1005-0957.2023.01.0056

[59] Xing YJ. Effect of fire-needle combined with abdominal acupuncture on patients with ankylosing spondylitis. Med J Chin Peoples Health. (2021) 33:64–6. doi: 10.3969/j.issn.1672-0369.2021.12.026

[60] Yang YX. Clinical observation of warm-needle moxibustion at Jiaji points in the treatment of 30 cases of ankylosing spondylitis. Guid J Tradit Chin Med Pharm. (2010) 16:88–9. doi: 10.13862/j.cnki.cn43-1446/r.2010.06.049

[61] Yuan WL. Clinical observation of governor-vessel–regulating and mind-soothing acupuncture for ankylosing spondylitis. J Pract Tradit Chin Med. (2022) 38:659–61. doi: 10.3969/j.issn.1004-2814.2022.4.syzyyzz202204075

[62] Guo CY Wang ZH Li L Li L Huang M Li SR. Clinical observation on patients with ankylosing spondylitis by bee acupuncture at Panlong acupoint and Taiyang Meridian Shu point. Yunnan J Tradit Chin Med Mater Medica. (2021) 42:58–61. doi: 10.16254/j.cnki.53-1120/r.2021.07.019

[63] Li L Yi XL Hou M Gang WS Yang CY Zhao MJ . Clinical observation of the sweat-inducing and Xuandu-opening method using acupuncture-cupping for 33 cases of ankylosing spondylitis. Chin J Tradit Med Sci Technol. (2015) 22:427–8.

[64] Li XH Wei S Chen ZH Hou CF. Clinical observation on minimally invasive tendon therapy and sulfasalazine in treating AS. West J Tradit Chin Med. (2020) 33:127–31. doi: 10.12174/j.issn.1004-6852.2020.05.36

[65] Lu ZS Cheng XL Ge LM Zheng YL. Effect of floating-needle therapy combined with reperfusion activity on ankylosing spondylitis. Guangming J Chin Med. (2024) 39:726–9. doi: 10.3969/j.issn.1003-8914.2024.04.030

[66] Luo YX Zhong DJ Huang YC Li ZP. Clinical observation on acupuncture in treating patients with sleep disorders in ankylosing spondylitis. J Guangzhou Univ Tradit Chin Med. (2024) 41:3210–5. doi: 10.13359/j.cnki.gzxbtcm.2024.12.019

[67] Meng L Yang GW Hou LQ. Effect of thalidomide combined with acupuncture on ankylosing spondylitis. Chin Foreign Med Res. (2022) 20:11–5. doi: 10.14033/j.cnki.cfmr.2022.13.004

[68] She RT Li WY Liu GK Lin YF. Clinical observation on acupuncture in treating patients with sleep disorders in ankylosing spondylitis. J Guangzhou Univ Tradit Chin Med. (2019) 36:1012–7. doi: 10.13359/j.cnki.gzxbtcm.2019.07.017

[69] Wang Y Liu YX Liu ZJ Ma YC. Observation of the effect of acupotomy in the treatment of ankylosing spondylitis. Clin Misdiagnosis Mistherapy. (2010) 23:712–3. doi: 10.3969/j.issn.1002-3429.2010.08.006

[70] Wang CY Li F. Observation of the efficacy of silver-needle therapy combined with medical ozone for ankylosing spondylitis with hip-joint stenosis. Henan Med Res. (2015) 24:51–2. doi: 10.3969/j.issn.1004-437X.2015.03.022

[71] Wang ZQ Wang LH Yi F Dong BQ Lin XX. Clinical effect of tendon acupuncture on patients with mild to moderate ankylosing spondylitis. J North Sichuan Med Coll. (2022) 37:22–6. doi: 10.3969/j.issn.1005-3697.2022.01.005

[72] Xie GS Wang QC Li CH. Clinical observation of the efficacy of silver-needle therapy for ankylosing spondylitis. J Anhui Tradit Chin Med Coll. (2009) 28:34–6. doi: 10.3969/j.issn.1000-2219.2009.02.013

[73] Zhan C Wu Q Ding Y Cai Y. Effect of internal-heat acupuncture on clinical efficacy and immune function in patients with ankylosing spondylitis. Lishizhen Med Mater Med Res. (2019) 30:2693–5. doi: 10.3969/j.issn.1008-0805.2019.11.044

[74] Zhang JL Liu XD Chen Y Liu FY. Effect of bee-venom therapy on peripheral blood TNF-α and IL-1β in ankylosing spondylitis. Zhejiang J Tradit Chin Med. (2010) 45:136–7. doi: 10.3969/j.issn.0411-8421.2010.02.045

[75] Zheng GX Huang RC. Clinical observation of bee-venom acupuncture combined with heat-sensitive moxibustion for ankylosing spondylitis. New Chin Med. (2015) 47:262–4. doi: 10.13457/j.cnki.jncm.2015.07.117

[76] Zheng X. Application effect of acupuncture and moxibustion in patients with ankylosing spondylitis. Clin Res Pract. (2020) 5:118–20. doi: 10.19347/j.cnki.2096-1413.202007050

[77] Zhou ZH Chen ZH Xu ZQ. Ankylosing spondylitis by warm needling on Jiaji point. J Clin Acupunct Moxibustion. (2011) 27:11–3. doi: 10.3969/j.issn.1005-0779.2011.03.004

[78] Wang XT Zheng SL Huang HQ Fang F Zhou TM. Effect of warm-needle moxibustion combined with spinal fixed-point repositioning on trunk function and inflammatory factors in patients with ankylosing spondylitis. Mod Pract Med. (2022) 34:313–5. doi: 10.3969/j.issn.1671-0800.2022.03.014

[79] Wu ZQ Su YH. Observation of the efficacy of combined acupuncture and moxibustion in 40 cases of ankylosing spondylitis. Heilongjiang J Tradit Chin Med. (2009) 38:41.

[80] Wang YJ Qiu WJ. Observations on the efficacy of superficial needling therapy for early ankylosing spondylitis. Shanghai J Acupunct Moxibustion. (2017) 36:1088–91. doi: 10.13460/j.issn.1005-0957.2017.09.1088

[81] Yang XY Guo J Chen ZR Zhao DC. Effect of acupotome on the expression of substance P, IL-6, IL-2 and TNF-a of ankylosing spondylitis patients. J Basic Chin Med. (2015) 21:723–4. doi: 10.19945/j.cnki.issn.1006-3250.2015.06.038

[82] Vickers AJ Vertosick EA Lewith G MacPherson H Foster NE Sherman KJ . Acupuncture for chronic pain: update of an individual patient data meta-analysis. J Pain. (2018) 19:455–74. doi: 10.1016/j.jpain.2017.11.005, 29198932 PMC5927830

[83] Fei YT Cao HJ Xia RY Chai QY Liang CH Feng YT . Methodological challenges in design and conduct of randomised controlled trials in acupuncture. BMJ. (2022) 376:e064345. doi: 10.1136/bmj-2021-064345, 35217507 PMC8868049

[84] Linde K Niemann K Schneider A Meissner K. How large are the nonspecific effects of acupuncture? A meta-analysis of randomized controlled trials. BMC Med. (2010) 8:75. doi: 10.1186/1741-7015-8-75, 21092261 PMC3001416

[85] Huang D Li Y Zheng X Hu J Tang H Yin Y . Acupuncture dosage and its correlation with effectiveness in patients with chronic stable angina: a systematic review and meta-analysis of randomized controlled trial. J Pain Res. (2025) 18:105–25. doi: 10.2147/JPR.S489880, 39811250 PMC11730283

[86] Vickers AJ Cronin AM Maschino AC Lewith G MacPherson H Foster NE . Acupuncture for chronic pain: individual patient data meta-analysis. Arch Intern Med. (2012) 172:1444–53. doi: 10.1001/archinternmed.2012.3654, 22965186 PMC3658605

[87] MacPherson H Altman DG Hammerschlag R Li Y Wu T White A . Revised standards for reporting interventions in clinical trials of acupuncture (Stricta): extending the consort statement. Acupunct Med. (2010) 28:83–93. doi: 10.1136/aim.2009.001370, 20615861 PMC3002761

[88] Hu X y Xiu W c Tian Z y Jiao R m Chen H Hu X y . Methodological challenges and recommendations for acupuncture clinical study: a scoping review. World J Acupunct Moxibustion. (2025) 35:1–9. doi: 10.1016/j.wjam.2024.12.008

[89] Ramiro S Nikiphorou E Sepriano A Ortolan A Webers C Baraliakos X . ASAS-EULAR recommendations for the management of axial spondyloarthritis: 2022 update. Ann Rheum Dis. (2023) 82:19–34. doi: 10.1136/ard-2022-223296, 36270658

[90] Liu S Wang Z Su Y Qi L Yang W Fu M . A neuroanatomical basis for electroacupuncture to drive the vagal–adrenal axis. Nature. (2021) 598:641–5. doi: 10.1038/s41586-021-04001-4, 34646018 PMC9178665

[91] Wang Y Chen Y Meng L Wu B Ouyang L Peng R . Electro-acupuncture treatment inhibits the inflammatory response by regulating γδ T and Treg cells in ischemic stroke. Exp Neurol. (2023) 362:114324. doi: 10.1016/j.expneurol.2023.114324, 36669751

[92] Rodolfi S Davidson C Vecellio M. Regulatory T cells in spondyloarthropathies: genetic evidence, functional role, and therapeutic possibilities. Front Immunol. (2024) 14:1303640. doi: 10.3389/fimmu.2023.1303640, 38288110 PMC10822883

[93] Benedetti G Miossec P. Interleukin 17 contributes to the chronicity of inflammatory diseases such as rheumatoid arthritis. Eur J Immunol. (2014) 44:339–47. doi: 10.1002/eji.201344184, 24310226

[94] Dou B Li Y Ma J Xu Z Fan W Tian L . Role of neuroimmune crosstalk in mediating the anti-inflammatory and analgesic effects of acupuncture on inflammatory pain. Front Neurosci. (2021) 15:695670. doi: 10.3389/fnins.2021.695670, 34408622 PMC8366064

[95] Sun SY Yan QQ Qiao LN Shi YN Tan LH Yang YS. Electroacupuncture alleviates pain responses and inflammation in collagen-induced arthritis rats via suppressing the TLR2/4-MyD88-NF-κB signaling pathway. Evid Based Complement Alternat Med. (2023) 2023:9050763. doi: 10.1155/2023/9050763, 36785752 PMC9922193

[96] Wei Y Dong M Zhong L Liu J Luo Q Lv Y . Regulation of hypothalamic-pituitary-adrenal axis activity and immunologic function contributed to the anti-inflammatory effect of acupuncture in the OVA-induced murine asthma model. Neurosci Lett. (2017) 636:177–83. doi: 10.1016/j.neulet.2016.11.001, 27816549

[97] Zheng J y Zhu J Wang Y Tian Z z. Effects of acupuncture on hypothalamic–pituitary–adrenal axis: current status and future perspectives. J Integr Med. (2024) 22:445–58. doi: 10.1016/j.joim.2024.06.004, 38955651

[98] Zhang RX Lao L Wang X Fan A Wang L Ren K . Electroacupuncture attenuates inflammation in a rat model. J Altern Complement Med. (2005) 11:135–42. doi: 10.1089/acm.2005.11.135, 15750372

[99] Wang S j Zhang J j Yang H y Wang F Li S t. Acupoint specificity on acupuncture regulation of hypothalamic-pituitary-adrenal cortex axis function. BMC Complement Altern Med. (2015) 15:87. doi: 10.1186/s12906-015-0625-4, 25887143 PMC4378553

[100] Xu TC Wen J Wang L Huang YY Zhu ZJ Zhu Q . Acupuncture indication knowledge bases: meridian entity recognition and classification based on ACUBERT. Database. (2024) 2024:baae083. doi: 10.1093/database/baae083, 39213389 PMC11363959

[101] Xiu W c Gang W j Zhou Q Shi L j Hu X y Ming T y . Factors and their impact on treatment effect of acupuncture in different outcomes: a meta-regression of acupuncture randomized controlled trials. Chin J Integr Med. (2024) 30:260–6. doi: 10.1007/s11655-023-3617-0, 38212500

[102] Zhang RF Zhou SN Bao YF Huo XN Fang Y Xu TC. Comparative analysis between new acupuncture and meridians and acupoints using the complex network approach. World J Acupunct Moxibustion. (2023) 33:150–4. doi: 10.1016/j.wjam.2022.05.003

[103] Shi M Hou B. Analysis of acupuncture selection rules for neuromyelitis optica spectrum diseases treated by acupuncture based on data mining. J Med Health Sci. (2025) 3:40–8. doi: 10.62517/jmhs.202505109

[104] Kroon FP Van Der Burg LR Ramiro S Landewé RB Buchbinder R Falzon L . Non-steroidal anti-inflammatory drugs (NSAIDs) for axial spondyloarthritis (ankylosing spondylitis and non-radiographic axial spondyloarthritis). Cochrane Database Syst Rev. (2015) 2015:CD010952. doi: 10.1002/14651858.CD010952.pub2, 26186173 PMC8942090

[105] Xiang ZD Zhang ZX Li C Du ZY. Investigation of efficiency and stability of cucumber mosaic virus-based gene silencing vector. Acta Agric Zhejiangensis. (2017) 29:625–30. doi: 10.3969/j.issn.1004-1524.2017.04.16

[106] Cao X Zhang Y Xiao Z Peng J. Efficacy and safety of acupuncture combined with Western medicine in the treatment of ankylosing spondylitis: a systematic review and meta-analysis. Medicine (Baltimore). (2025) 104:e42468. doi: 10.1097/md.0000000000042468, 40419885 PMC12113976

[107] Triantafyllias K Sauer C Schwarting A. Therapeutic effects of complex multimodal rheumatologic treatment in the rheumatology center, Rhineland-Palatinate. Z Rheumatol. (2022) 81:596–604. doi: 10.1007/s00393-022-01209-1, 35532799

[108] Wan R Ji Y Fan Y Yang C Yang H Gou X . Efficacy and safety of Duhuo Jisheng decoction combined with Western medicine in the treatment of ankylosing spondylitis: a systematic review and meta-analysis. Complement Ther Clin Pract. (2023) 51:101739. doi: 10.1016/j.ctcp.2023.101739, 36809734

