# Supplementary material for: A systematic review and meta-analysis of the effect of acupuncture therapy on the symptoms and immune indicators of ankylosing spondylitis
Source: Front Neurol. 2026 Jan 12;16:1652356. doi: 10.3389/fneur.2025.1652356 (PMC12833089; doi:10.3389/fneur.2025.1652356)
Supplement: Supplementary file 2 [file Table_2.docx]

**English database**

1. Pubmed

| Pubmed | |
| --- | --- |
| #1 | Search:(Ankylosing spondylitis [MeSH Terms]) OR (Spondylitis,Ankylosing [Title/ Abstract] OR Ankylosing spondylitis[Title/ Abstract]OR Ankylosing Spondylarthritis [Title/ Abstract] OR AnkylosingSpondylarthritides [Title/ Abstract] OR Spondylarthritides |
| #2 | Search:(Acupuncture [MeSH Terms]) OR (Acupuncture [Title/ Abstract]  OR electroacupuncture [Title/ Abstract] OR electro-  Acupuncture [Title/ Abstract] OR electric acupuncture [Title/ Abstract] OR acupoint [Title/ Abstract] OR acupoints [Title/ Abstract] ORAcupotomy [Title/ Abstract]) |
| #3 | #1 AND #2  Search: ((Ankylosing spondylitis [MeSH Terms]) OR (Spondylitis,Ankylosing[Title/ Abstract] OR Ankylosing spondylitis [Title/ Abstract] OR Ankylosing Spondylarthritis [Title/ Abstract] OR AnkylosingSpondylarthritides[Title/ Abstract] OR Spondylarthritides,  Ankylosing [Title/ Abstract] OR Spondylarthritis,  Ankylosing [Title/ Abstract] OR Ankylosing  Spondyloarthritis [Title/ Abstract] OR Ankylosing  Spondyloarthritides [Title/ Abstract] OR  Spondyloarthritides, Ankylosin [Title/ Abstract] OR Spondyloarthritis,  Ankylosing [Title/ Abstract] OR Spondylitis  Ankylopoietica [Title/ Abstract] OR Bechterew Disease [Title/ Abstract] OR Spondyloarthritis Ankylopoietica [Title/ Abstract])) AND ((Acupuncture [MeSH Terms]) OR (Acupuncture [Title/ Abstract] OR  Electroacupuncture [Title/ Abstract] OR electro-  acupuncture [Title/ Abstract] OR electric acupuncture [Title/ Abstract] OR acupoint [Title/ Abstract] OR acupoints [Title/ Abstract] OR Acupotomy[Title/ Abstract])) |

1. Web of Science

| Web of Science | |
| --- | --- |
| #1 | Spondylitis, Ankylosing or Ankylosing spondylitis or Ankylosing Spondylarthritis or Ankylosing Spondylarthritides or Spondylarthritides, Ankylosing or Spondylarthritis, Ankylosing or Ankylosing Spondyloarthritis or Ankylosing Spondyloarthritides or Spondyloarthritides,Ankylosin or Spondyloarthritis, Ankylosing or Spondylitis Ankylopoietica or Bechterew Disease or Spondyloarthritis Ankylopoietica |
| #2 | Acupuncture or electroacupuncture or electro-acupuncture or electric acupuncture or acupoint or acupoints or Acupotomy |
| #3 | #1 AND #2  Spondylitis, Ankylosing or Ankylosing spondylitis or Ankylosing Spondylarthritis or Ankylosing Spondylarthritides or Spondylarthritides, Ankylosing or Spondylarthritis, Ankylosing or Ankylosing Spondyloarthritis or Ankylosing Spondyloarthritides or Spondyloarthritides,Ankylosin or Spondyloarthritis, Ankylosing or Spondylitis Ankylopoietica or Bechterew Disease or Spondyloarthritis Ankylopoietica (Topic) and Acupuncture or electroacupuncture or electro-acupuncture or electric acupuncture or acupoint or acupoints or Acupotomy (Topic) |

1. Cohrane

| Cohrane | |
| --- | --- |
| #1 | MeSH descriptor: [Spondylitis, Ankylosing] explode all trees |
| #2 | Title Abstract Keyword: (Spondylitis, Ankylosing or Ankylosing spondylitis or Ankylosing Spondylarthritis or Ankylosing Spondylarthritides or Spondylarthritides, Ankylosing or Spondylarthritis, Ankylosing or Ankylosing Spondyloarthritis or Ankylosing Spondyloarthritides or Spondyloarthritides,Ankylosin or Spondyloarthritis, Ankylosing or Spondylitis Ankylopoietica or Bechterew Disease or Spondyloarthritis Ankylopoietica):ti,ab,kw (Word variations have been searched) |
| #3 | #1 OR #2 |
| #4 | MeSH descriptor: [Acupuncture] explode all trees |
| #5 | Title Abstract Keyword: (Acupuncture or electroacupuncture or electro-acupuncture or electric acupuncture or acupoint or acupoints or Acupotomy):ti,ab,kw (Word variations have been searched) |
| #6 | #4 OR #5 |
| #7 | #3 AND #6 |

1. Embase

| Embase | |
| --- | --- |
| #1 | 'spondylites, ankylosing's ab kw OR 'ankriosina spondylitis"t.ab ke OR 'ankyiosing spondylarthritis" ti,ab.kw OR 'ankylosing spondylarthritides" ab.kw OR 'spondylarthritides. ankylosing' ti,ab.kw OR 'spondylarthritis, ankylosing' ti,ab.kw OR "ankylosing spondyloarthritis' ti,ab.kw OR 'ankylosing spondyloarthritides' ti.ab.kw OR spondyloarthritides ankylosin ti,ab.kw OR'spondylearthritis, ankylosing’ ti,ab.kw OR 'spandylitis ankylopoietica' ti,ab.kw OR 'spondyloarthritis ankyiopoietica' ti,ab.kw OR 'ankylosing spondylitis'/ exp |
| #2 | acupuncture ti, ab, kw OR electroacupuncture ti, ab, kw OR ' electroacupuncture' ti, ab, kw OR' electric acupuncture' ti, ab, kw OR acupoint ti, ab, kw OR acupoints: ti, ab, kw OR acupotomy: ti, ab, kw OR' acupuncture'/ exp |
| #3 | #1 AND #2 |

**Chinese database**

1. CNKI

| CNKI | |
| --- | --- |
| #1 | 主题：强直性脊柱炎 + 脊柱炎 + 强直性 + 大偻 + 骨痹 |
| #2 | 主题：针灸 + 针刺 + 温针灸 + 雷火针灸 + 电针 + 针法 + 穴位 + 针刀 + 选穴 + 耳针 |
| #3 | 主题：临床随机对照试验 + 临床观察 + 疗效 + 临床研究 + 机制 + 通路 + 分子 + 因子 + 动物 |
| #4 | #1 AND #2 AND #3  （主题：强直性脊柱炎 + 脊柱炎 + 强直性 + 大偻 + 骨痹）AND（主题：针灸 + 针刺 + 温针灸 + 雷火针灸 + 电针 + 针法 + 穴位 + 针刀 + 选穴 + 耳针）AND（主题：临床随机对照试验 + 临床观察 + 疗效 + 临床研究） |

1. Wanfang

| Wanfang | |
| --- | --- |
| #1 | 主题:("强直性脊柱炎 OR 脊柱炎 OR 强直性 OR 大偻 OR 骨痹") or 主题:("脊柱炎") or 主题:("强直性") or 主题:("大偻") or 主题:("骨痹") |
| #2 | 主题:(针灸) or 主题:(针刺 ) or 主题:(温针灸) or 主题:(雷火针灸) 主题:( 电针) or 主题:( 针法) or 主题:(穴位) or 主题:(针刀) or 主题:(选穴 ) or 主题:(耳针) |
| #3 | 主题:(临床随机对照试验) or 主题:(临床观察) or 主题:(疗效) or 主题:(临床研究) |
| #4 | #1 AND #2 AND #3   (主题:("强直性脊柱炎 OR 脊柱炎 OR 强直性 OR 大偻 OR 骨痹") or 主题:("脊柱炎") or 主题:("强直性") or 主题:("大偻") or 主题:("骨痹")) AND (主题:(针灸) or 主题:(针刺 ) or 主题:(温针灸) or 主题:(雷火针灸) 主题:( 电针) or 主题:( 针法) or 主题:(穴位) or 主题:(针刀) or 主题:(选穴 ) or 主题:(耳针)) AND (主题:(临床随机对照试验) or 主题:(临床观察) or 主题:(疗效) or 主题:(临床研究) ) |

1. VIP

| VIP | |
| --- | --- |
| #1 | 主题：强直性脊柱炎 OR 脊柱炎 OR 强直性 OR 大偻 OR 骨痹 |
| #2 | 主题：针灸 + 针刺 + 温针灸 + 雷火针灸 + 电针 + 针法 + 穴位 + 针刀 + 选穴 + 耳针 |
| #3 | 主题：临床随机对照试验 + 临床观察 + 疗效 + 临床研究 |
| #4 | #1 AND #2 AND #3  主题=强直性脊柱炎 OR 脊柱炎 OR 强直性 OR 大偻 OR 骨痹 AND 主题=针灸 + 针刺 + 温针灸 + 雷火针灸 + 电针 + 针法 + 穴位 + 针刀 + 选穴 + 耳针 AND 主题=临床随机对照试验 + 临床观察 + 疗效 + 临床研究 |

1. Sinomed

| Sinomed | |
| --- | --- |
| #1 | 常用字段：强直性脊柱炎 OR 脊柱炎 OR 强直性 OR 大偻 OR 骨痹 |
| #2 | 常用字段：针灸 OR 针刺 OR 温针灸 OR 雷火针灸 OR 电针 OR 针法 OR穴位 OR针刀 OR 选穴 OR 耳针 |
| #3 | 常用字段：临床随机对照试验 OR 临床观察 OR 疗效 OR 临床研究 |
| #4 | #1 AND #2 AND #3  [( "强直性脊柱炎"[常用字段:智能] OR "脊柱炎"[常用字段:智能] OR "强直性"[常用字段:智能] OR "大偻"[常用字段:智能] OR "骨痹"[常用字段:智能]) AND( "针灸"[常用字段:智能] OR "针刺"[常用字段:智能] OR "温针灸"[常用字段:智能] OR "雷火针灸"[常用字段:智能] OR "电针"[常用字段:智能] OR "针法 OR穴位 OR针刀"[常用字段:智能] OR "选穴"[常用字段:智能] OR "耳针"[常用字段:智能]) AND( "临床随机对照试验"[常用字段:智能] OR "临床观察"[常用字段:智能] OR "疗效"[常用字段:智能] OR "临床研究"[常用字段:智能])](javascript:this.top.vpn_inject_scripts_window(this);vpn_eval((function%20()%20%7b%20toDoRelimitSearch();%20%7d).toString().slice(14,%20-2))) |
